# *Trypanosoma cruzi* in the Chicken Model: Chagas-Like Heart Disease in the Absence of Parasitism

**DOI:** 10.1371/journal.pntd.0001000

**Published:** 2011-03-29

**Authors:** Antonio R. L. Teixeira, Clever Gomes, Nadjar Nitz, Alessandro O. Sousa, Rozeneide M. Alves, Maria C. Guimaro, Ciro Cordeiro, Francisco M. Bernal, Ana C. Rosa, Jiri Hejnar, Eduardo Leonardecz, Mariana M. Hecht

**Affiliations:** 1 Chagas Disease Multidisciplinary Research Laboratory, Faculty of Medicine, University of Brasilia, Brasilia, Federal District, Brazil; 2 Laboratory of Viral and Cellular Genetics, Institute of Molecular Genetics, Academy of Sciences of the Czech Republic, Prague, Czech Republic; New York University School of Medicine, United States of America

## Abstract

**Background:**

The administration of anti-trypanosome nitroderivatives curtails *Trypanosoma cruzi* infection in Chagas disease patients, but does not prevent destructive lesions in the heart. This observation suggests that an effective treatment for the disease requires understanding its pathogenesis.

**Methodology/Principal Findings:**

To understand the origin of clinical manifestations of the heart disease we used a chicken model system in which infection can be initiated in the egg, but parasite persistence is precluded. *T. cruzi* inoculation into the air chamber of embryonated chicken eggs generated chicks that retained only the parasite mitochondrial kinetoplast DNA minicircle in their genome after eight days of gestation. Crossbreeding showed that minicircles were transferred vertically via the germ line to chicken progeny. Minicircle integration in coding regions was shown by targeted-primer thermal asymmetric interlaced PCR, and detected by direct genomic analysis. The kDNA-mutated chickens died with arrhythmias, shortness of breath, cyanosis and heart failure. These chickens with cardiomyopathy had rupture of the dystrophin and other genes that regulate cell growth and differentiation. Tissue pathology revealed inflammatory dilated cardiomegaly whereby immune system mononuclear cells lyse parasite-free target heart fibers. The heart cell destruction implicated a thymus-dependent, autoimmune; self-tissue rejection carried out by CD45^+^, CD8γδ^+^, and CD8α lymphocytes.

**Conclusions/Significance:**

These results suggest that genetic alterations resulting from kDNA integration in the host genome lead to autoimmune-mediated destruction of heart tissue in the absence of *T. cruzi* parasites.

## Author Summary

The *Trypanosoma cruzi* acute infections can be asymptomatic but approximately one third of the chronically infected cases may present Chagas disease. Parasite persistence and autoimmunity are theories trying to explain the clinical and pathological manifestations of Chagas disease in the heart and the digestive system. To clearly demonstrate roles played by parasite persistence and autoimmunity in Chagas disease we used a chicken model refractory to the *T. cruzi*. In this study we inoculated the invasive *T. cruzi* in the air chamber of embryonated eggs. The infection was eradicated by the innate immunity and the chicks were parasite-free at hatching, but they retained the parasitic mitochondrial kinetoplast DNA minicircle in their genome. We documented the kDNA minicircle integrated in the chicken genome by a targeted prime TAIL-PCR, Southern hybridizations, cloning and sequencing. The kDNA minicircles integrated in coding regions of various chromosomes, and mutated chickens developed an inflammatory cardiomyopathy hallmark of Chagas disease, whereby immune system mononuclear cells lyse parasite-free target heart fibers. Genotype alterations resulting from transfers of the parasitic DNA were associated with the tissue destruction carried out by effectors CD45^+^, CD8γδ^+^, CD8α lymphocytes. This research provides insights about a protozoan infection that can induce genetically driven autoimmune disease.

## Introduction

*Trypanosoma cruzi* infection (American Trypanosomiasis) is an endemic ailment transmitted by hematophagous (Reduviid:Triatominae) bugs, by blood transfusion and transplacentally from the mother to offspring [1]. In pregnant women *T. cruzi* infections may lead to fetal complications, with desorption of the embryo, stillbirth, neonatal death, intrauterine growth retardation, or prematurity [2]–[7]. These infections are highly prevalent in rural areas of Latin America, where an estimated 18 million people harbor *T. cruzi*, and over 100 million are at risk of acquisition [8]. The migration of *T. cruzi*-infected patients from endemic areas has made Chagas disease cosmopolitan, now emerging in five continents as an important global health problem requiring specific training of personnel for diagnosis and delivery of medical assistance [9].

The acute *T. cruzi* infections are usually asymptomatic and go unrecognized, but high rates of morbidity and lethality are recorded in chronically infected cases [1], [10], [11]. Chagas disease is a multifaceted clinical condition encountered in approximately one third of the human population with *T. cruzi* infections; the disease attacks the heart in 94.5% of cases, and the esophagus and/or the colon (mega syndromes) in 5.5% of the chronically infected individuals. The hallmark of the disease is a destructive myocarditis [10], which typically is lethal two to five years after presenting signs of impairment of blood circulation [11].

The administration of an anti-trypanosome nitroderivative to treat human *T. cruzi-*infections did not prevent destructive heart lesions and death [12], [13], thus an effective treatment for Chagas disease requires further knowledge about parasite-host relationships and its pathogenesis [1]. Two theories are proposed to explain the pathogenesis of Chagas disease: *i*) Parasite persistence with rupture of parasitized cells and release of parasitic antigens that attracts inflammatory cells infiltrates [14], [15]; and *ii*) Autoimmune rejection of target cells by the immune system inflammatory effector cells [1], [16], [17]. The second hypothesis is difficult to test, because other mechanisms of tissue inflammation may coexist in the setting of an active infection [18], [19]. On the one hand, the cryptic infections are sources of parasitic antigens and inflammation, or they persist for decades without causing the host significant damage? On the other, a clear demonstration of the part autoimmunity plays on the development of Chagas heart disease is essential for the effective delivery of treatment.

Upon entry of *T. cruzi* into the body, the infective trypomastigote form can be destroyed by the monocyte-macrophage system, but internalized parasites in non-phagocyte cells can replicate as amastigotes before returning to trypomastigotes that then emerge, invading any tissue or cell type. The *T. cruzi* genome measures 60.3 Mbp [20], [21], and its total DNA ranges from 125–280 fg/cell [22]–[24]. Those broad differences are explained by relative chromosome number and size due to insertions, duplications and deletions, or by the relative contents of haploid, diploid or aneuploid cells during the growth process [25]. *T. cruzi* has a unique mitochondrion with a large amount of extranuclear DNA (kDNA) that can reach 15% to 30% of total cellular DNA [26], which differs from the nuclear component by buoyant density, base ratio, and degree of renaturation [27]. A kDNA network is composed of catenated rings with a few dozen maxicircles (20 to 40 kb) and thousands of minicircles (1.4 kb). The maxicircles are structurally and functionally analogous to the mitochondrial DNA in higher eukaryotes, encoding rRNAs and subunits of the respiratory complexes [28], [29]. Topologically, each *T. cruzi* minicircle has four average 240 bp hypervariable regions interspersed by 122 bp conserved regions each of which presents conserved cytosine/adenine-rich sequence blocks (CArsbs) [29]–[33]. It is through sequence microhomologies in the CArsbs that foreign DNA integration is thought to occur [34]. The minicircles encode guide RNAs (gRNAs), which modify the maxicircle transcripts by extensive uridine insertion or deletion, in a process known as RNA editing. Information for this process is provided by small gRNA molecules encoded primarily on the kDNA minicircles. The unusual organization of kinetoplastid genes in directional gene clusters requires equally unorthodox mechanisms to generate functional eukaryotic mRNA [28, 35, and 36]. The sequence heterogeneity of the thousands of kDNA minicircles in each cell represents an additional layer of complexity, thus augmenting genetic diversity.

Horizontal transfer of kDNA minicircle sequences into the genome of *T. cruzi-*infected macrophages and of chagasic rabbits and humans is documented [34], [37]–[39]. *T. cruzi* minicircle sequences integrated mainly in retrotransposable elements present in chromosomes of rabbits and of people with *T. cruzi* infections. Subsequent recombination and hitchhiking propagate minicircle sequence insertions in coding regions, rupturing open reading frames or knocking out genes in the host genome [1], [10], [34], [37]–[39]. In a broad sense, the demonstration of kDNA minicircle sequences integrated into the genome of mammalians could be challenged by the possibility of contamination with DNA residues of the *T. cruzi* life long infections in susceptible hosts. Therefore, to further document possible roles played by LkDT-induced genotype alteration in the proposed autoimmune pathogenesis of Chagas disease it was required the live infection to be omitted. This important requirement could be fulfilled in the crosskingdom chicken model system that would eradicate the *T. cruzi* infections.

The chicken genome has a haploid content of 1.2×10^9^ bp (20,000–23,000 genes) divided among 39 chromosomes. Autosomes are classified into macrochromosomes 1 through 5, intermediate chromosomes 6 through 10, and microchromosomes 11 through 32. The sex chromosomes are denominated Z and W, with homogametic males (Z/Z) and heterogametic females (Z/W). Repetitive elements make up 10% of the chicken genome, compared with 40–50% in the genomes of most mammals. A relatively compact genome structure is the result of the limited accumulation of repetitive elements. Unlike other vertebrate genomes, active short interspersed nuclear elements (SINEs) are not found in the chicken genome. Most retroelements are found in G+C-rich regions and many of the chicken repeats-1 (CR-1) flank multiple genes, but CR-1 elements may also accumulate within A+T rich satellite regions [40], [41].

In this study we describe an experimental crosskingdom host model for parasite-free heart disease in chickens, which are refractory to *T. cruzi* infection [42] except during early embryonic life, prior to the development of their immune system [10]. Chicks hatched from *T. cruzi*-infected eggs retained minicircle sequences in the absence of parasite nuclear DNA (nDNA). Moreover we document integration of kDNA into the DNA of somatic and germ line cells, from where they are vertically transmitted to subsequent progeny. kDNA mutations were detected mainly in coding regions on several chromosomes. Interestingly, kDNA-mutated chickens developed gross cardiomegaly with an inflammatory myocarditis similar to that of Chagas disease in man, in which parasite-free myofibers are destroyed by immune system effector cells, as well as heart failure.

## Methods

### Animals

White Ross chicken eggs were obtained from Asa Alimentos (Recanto das Emas, Federal District, Brazil). Chicks and adult birds were housed in the Faculty Animal Facility at room temperature. Protocols for all animal studies were approved by the Institutional Ethical Committee in Animal Research in accordance with international guidelines.

### Growth of parasites

Trypomastigotes forms of *T. cruzi* Berenice and the β-galactosidase-expressing Tulahuen *T. cruzi* MHOM/CH/00 C4 were used [43]. Trypomastigote forms of *T. cruzi* were grown in murine muscle cell (L6) cultivated in Dulbecco minimal essential medium with 10% FSB, 100 IU/ml penicillin, 100 µg/ml streptomycin, and 250 nM L-glutamin (pH 7.2), 5% CO_2_ at 37°C. Epimastigote forms were grown in liver-infusion tryptose axenic medium at 27°C. The parasite forms were harvested at exponential growth phase.

### *Trypanosoma cruzi* inoculation in embryonated chicken eggs

In the test group, a 2-mm diameter hole pierced in the shell of 60 fertile eggs for injecting with 100 forms of *T. cruzi* trypomastigotes in 10 µL of culture medium into the air chamber of stage X embryos. In the control group, 36 mock chickens received 10 µL of culture medium alone. Holes were sealed by adhesive tape, and the *T. cruzi-*infected eggs as well mock and 12 uninfected control samples were incubated at 37.5°C and 65% humidity for 21 days. The viable embryos (86%) initiated growth upon incubation, and after 21 days the chicks that hatched were kept in incubatory for 24 h and thereafter at 32°C for three weeks.

### Obtaining samples for DNA extraction

The peripheral blood mononuclear cells and solid tissues from 48 kDNA-mutated and from 22 control and mock (shells pierced but not *T. cruzi* or kDNA inoculated) chickens were processed for DNA extraction. DNA was also extracted from semen collected from roosters, and from nonfertilized eggs (<5 mm) from hens hatched from fertile eggs inoculated with *T. cruzi* [44]. The mitochondrial kDNA was obtained from *T. cruzi* epimastigote forms as described elsewhere [45].

### Primers and probes used

The primers used for PCR amplifications and the thermal conditions are shown in **Table 1**. The probes used in Southern blot hybridizations were: 1) Wild-type kDNA (∼1.4 kb) minicircle sequences purified from *T. cruzi* epimastigote forms; 2) kDNA minicircle fragments (362 bp) obtained by *Nsi*I digests of wild-type kDNA; and 3) nDNA repetitive sequence (188 bp) obtained by amplification of the parasite DNA with the Tcz1/2 primers. The probes were purified from 1% agarose gels.

**Table 1:** Probes used in the *tp*TAIL-PCR amplifications.

Primer	Target DNA	Sequence	Tm*
S 34	*T. cruzi* kDNA	5′ ACA CCA ACC CCA ATC GAA CC 3′	57,9
S 67	*T. cruzi* kDNA	5′ GGT TTT GGG AGG GG(G/C) (G/C)(T/G)T C 3′	60,1
S 35	*T. cruzi* kDNA	5′ ATA ATG TAC GGG (T/G)GA GAT GC 3′	59,4
S 36	*T. cruzi* kDNA	5′ GGT TCG ATT GGG GTT GGT G 3′	57,9
*Gg*1	*G. gallus*	5′ AGC TGA TCC TAA AGG CAG AGC 3′	60.1
*Gg2*	*G. gallus*	5′ CTG AGC CTC TGC TTT GAA A 3′	56.8
*Gg3*	*G. gallus*	5′ TTT CAA AGC AGA GGC TCG G 3′	60.1
*Gg4*	*G. gallus*	3′ GCT CTG CCT TTA GGA TCA GCT 5′	64.2
*Gg5*	*G. gallus*	3′ AGC AAC TCA GCG TCC ACC TT 5′	62.3
*Gg6*	*G.gallus*	3′ CTG TTA GCA TGA GGC TTC ACA A 5′	60.4
XeCRs-1^a^	*G. gallus*	5′ ATW TCW GTS TTT GCA GAT GAC ACA 3′	60.4
XeCRs-2	*G. gallus*	5′ CTT WGT TGC CCT YCT CTG KAC YCT CTC YA 3′	66.6
XeCRs-3	*G. gallus*	5′ TGT GTC ATC TGC AAA SAC WGA WAT 3′	65.3
XeCRs-4	*G. gallus*	5′TRG AGA GRG TMC AGA GRA GGG CAA CWA TG	3′ 67.9

*Tm  =  average annealing temperature °C.

a XeCrs primer sets were a gift from Professor Dusan Kordis to Dr. Jiri Hejnar, Czech Academy of Sciences, Praha.

### Southern blot and PCR analyses

Genomic DNAs from infected chicks and uninfected controls were templates for PCR with specific *T. cruzi* nDNA Tcz1/2 [46] and kDNA primers s35/s36 [47]. The standard PCR procedure consisted in using 100 ng template DNA, 0.4 µM of each pair of primers, 2 U Taq DNA polymerase, 0.2 mM dNTP and 1.5 mM MgCl_2_ in a 25 µL final volume. The sensitivity of Tcz1/2 primers was determined in a mix of 200 ng chicken DNA with serial dilutions of *T. cruzi* DNA (from 1 ng to 1 fg) and the standard procedure was carried out with same concentrations of reagents used in test experiments with chicken DNA alone. The temperature used was 95 °C for 5 min, 30 cycles of 30 secs at 95 °C/30 secs at 68 °C/1 min at 72 °C with 5 min final extension before refrigeration. The amplification products were analysed in 1.3% agarose gel, transferred to a positively-charged nylon membrane (GE Life Sciences) by the alkaline method for hybridization with specific probes labeled with [α-^32^P] dATP using Random Primer Labeling Kit (Invitrogen, Carlsbad, CA).

Southern hybridizations were performed with *Mbo*I and/or with *Eco*RI (Invitrogen) digests of DNA samples of body tissues from uninfected control chickens and from chickens hatched from eggs inoculated with virulent *T. cruzi* forms. The enzymes used made single cuts in minicircles. The digests of DNA from *T. cruzi* were subjected to electrophoresis in 0.8% agarose gel at 50 V overnight at 4°C. The gel transferred to positively charged nylon membrane was hybridized with radio labeled kDNA probe. The membrane was washed twice for 15 min at 65 °C with 2X SSC and 0.1% SDS, twice for 15 min at 65 °C each with 0.2X SSC and 0.1% SDS, and autoradiograph for variable periods of time.

### 5′ RACE and sequencing

The identification of *T. cruzi* kDNA minicircle integrated into the chicken genome was first shown using a standard protocol for 5′-RACE [48]. The amplification products were cloned directly in pGEM T Easy vector. The clones confirmed by DNA hybridization with a radioactively labeled wild-type kDNA probe were sequenced commercially (AY237306, FN600577).

### *tp*TAIL-PCR, validation of the *tp*TAIL-PCR, cloning and sequencing

A modification of the TAIL-PCR technique was used [34], [49], which combined kDNA primers with primer sets obtained after alignment of chimera sequence AY237306 within the loci NW_001471687.1 at the *G. gallus* genome. In the first round of amplifications, each reaction included 200 ng template DNA, 2.5 mM MgCl_2_, 0.4 µM of kDNA primers (S34 or S67), 0.2 mM dNTPs, 2.5 U *Taq* Platinum (Invitrogen, Carlsbad, CA). The kDNA primers were used in combination with 0.04 µM of *Gg* primers (*Gg*1 to *Gg*6, **Table 1**), separately. The targeting primers annealing temperatures ranged from 57.9 to 60.1 °C for kDNA primers, and from 59.9 to 65.6 °C for CR-1 primer sets (**Table 1**). These temperatures are higher than those (∼45 °C) required for the arbitrary degenerated primers used in the TAIL-PCR [49]. The temperature and cycles used (MyCycle Termocycler, Bio-Rad Laboratories, Hercules, CA) are described in a previous paper (34). In the second round of amplifications, PCR products were diluted 1∶40 (v/v) in water. kDNA primers S35 and S35 antisense were substituted for the nested ones, along with the same *Gg* primers. In the third step, PCR products of *tp*TAIL-PCR 2 were diluted 1∶10 (v/v) in water and the *Gg* primers were combined in the reaction with S67 antisense or S36, separately. PCR products of the last amplification that hybridize with kDNA probe were cloned directly in pGEM T easy vector (Promega, Madison, WI). Clones selected by hybridization with kDNA probe were sequenced commercially. The validation of the *tp*TAIL-PCR was determined in a mix of 300 pg of kDNA from *T. cruzi* with 200 ng of DNA from control birds never exposed to kDNA. The temperature and amplification cycles were the same used for the test birds' DNA.

### Chagas disease clinic manifestation

Growth and development of chickens hatched from *T. cruzi* infected eggs and of healthy controls hatched from non-infected eggs were monitored daily for mortality and weekly for disease manifestations. Clinical abnormalities in those chickens were detected by inspection and by disclosure of arrhythmias and of increasing heart size by electrocardiograph (ECG) recordings. A one-channel model apparatus was used for ECG recordings with standard 1 mV/cm and speed of 25 mm/sec. The electrodes were placed under the wing pits and on the back of the legs after removal of feathers and skin cleansing with the chicken in supine position. Chickens were submitted monthly to ECG recordings of frontal leads AVR, AVL and AVF, and assessment of deviation of mean electrical axis to the left, which is suggestive of heart enlargement, were obtained. The ECG recordings allowed evaluation of mean electrical axes, heart rates and arrhythmias. These experiments included equal number of control chickens for comparison.

### Pathology and immunochemical analyses

Heart and body weight indexes were obtained after natural deaths of kDNA-mutated chickens. For each experimental case, a control (kDNA- the negative) chicken of the same age and gender was sacrificed, and the heart weight (g)/body weight (kg) indexes were obtained. Tissues removed from the heart, esophagus, intestines, skeletal muscle, lungs, liver, and kidneys were fixed in buffered 10% formalin (pH 7.4), embedded in paraffin and cut to 4 µm thick sections for histological analyses after Hematoxylin-Eosin (HE) staining. Tissues that were harvested from embryos and from chicks at set times were bisected so that half could be fixed in 0.02% glutaraldehyde prepared in phosphate buffered saline (pH 7.2) and stained with X-Gal (43). X-Gal-stained tissues were then fixed in paraformaldehyde. Paraffin embedded tissues sections were mounted by standard methods for microscopic examination. Sections showing blue cells were subjected to incubation with a human Chagas diseased antiserum with specific anti-*T. cruzi* antibody 1∶1024 [12] and immunofluorescent staining with a fluorescein-conjugated rabbit anti-human IgG for colocalizing embryo cells harboring *T. cruzi.*

### Phenotyping immune system cells in heart lesions

Tissue sections of heart from kDNA-positive and from control kDNA-negative chickens were separated for phenotype immune effectors cells. The slides embedded in paraffin were placed at 65°C for 30 min to melt wax previous to submission to four baths in 100% to 70% xylene and then in absolute ethanol PBS for 5 min each. The slides rinsed in distilled water were air dried treated with the following antibodies: 1) Mouse anti-chicken Bu-1 (Bu-1^a^ and Bu-1^b^ alleles, Mr 70–75 kDa) Mab AV20 recognizing monomorphic determinant on the B cell antigens of inbred chickens. 2) Mouse anti-chicken CD45, Ig isotype IgM1κ specific to chicken thymus lineage cells (Mr 190 to 215-KDa variant). 3) Mouse anti-chicken TCRγδ (Mr 90-kDa heterodimer) Mab specific to thymus dependent CD8^+^γδ T cells. 4) Mouse anti-chicken Mab CT-8 specific to chicken α chain (Mr 34 kDa) recognizing the CD8 cells in thymocytes, spleen and peripheral blood. 5) Mouse anti-chicken KuL01 exclusively recognizing monocytes/macrophages of the phagocyte system. The monoclonal antibodies were fluorescein- or R-phycoerythrin-conjugate obtained from SouthernBiotech, Birmingham, AL. After incubation with specific anti-phenotype antibody the slide was washed three times with O.1 M PBS, pH 7.4, 5 min each. At the end the slide was washed trice with PBS and assembled with buffered glycerin for exam under a fluorescent light microscope with emission filter of wavelength 567 and 502 nm, respectively, to detect red and green fluorescence-labeled cells.

### Data analyses

The chicken genome database (http://www.ncbi.nlm.nih.gov/genome/seq/BlastGen/BlastGen.cgi?taxid=9031) was used for BLASTn sequence analyses. CLUSTALW alignments were performed and statistical significance (p<0.001) was determined for scores (e-values) recorded. The GIRI repeat masking algorithm CENSOR (http://girinst.org/censor/index.php) was employed for localization of different classes of repeats in chimeric sequences. The Kinetoplastid Insertion and Deletion Sequence Search Tool (KISS) were employed to identify potential gRNAs in the kDNA sequences [50]. The KISS database comprises *Trypanosoma brucei* and *Leishmania tarentolae* minicircle and maxicircle as well as a work bench for RNA editing analysis in kinetoplastids [50], [51] with the aid of WU-Blastn-modified-matrix [52]. Also, *T. cruzi* sequences (http://www.biomedcentral.com/content/supplementary/1471-2164-8-133-s1.fas) were used to search-in gRNAs in the kDNA-host DNA chimera sequences. Student's *t* test was used to detect significant differences between deviations of electric axes in the ECG tracings in kDNA-positive and in control healthy chickens, and between heart/body weight indexes obtained in the experimental and control groups. The Kolmorov-Smirnov test was used to detect mortality ratios significant differences between groups of chickens hatched from *T. cruzi* inoculated eggs and from mock control.

## Results

### *Trypanosoma cruzi* short run infections in embryonated chicken eggs and parasite-free inflammatory cardiomyopathy

**Figure 1:**
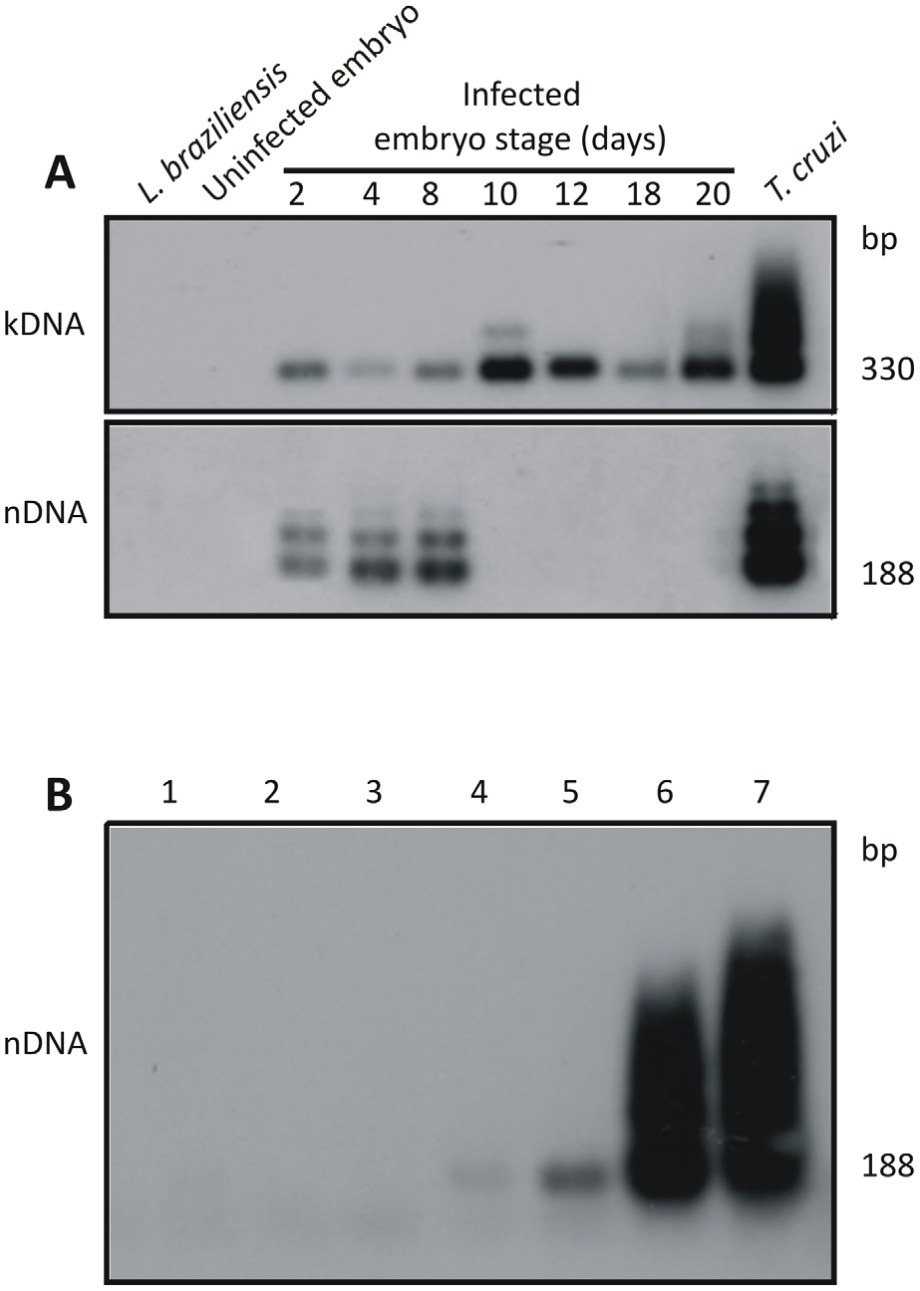
Elimination of *Trypanosoma cruzi* infection early in *Gallus gallus* embryonic development. A) Top panel shows 330 bp bands formed by PCR amplified minicircles kDNA templates harvested at several stages of the chicken embryonic development, after hybridization with a specific probe; Bottom panel shows bands formed by PCR amplified from same embryos after separation in 1% agarose gel and hybridization with a specific nDNA probe; the 188 bp nDNA band was diagnostic of the parasite persistence in the host tissue. B) Sensitivity of the PCR with nDNA primers Tcz1/2. Lanes 1 and 2, control DNA from kDNA negative and from kDNA-mutated chickens; Lanes 3 to 7, mix of 200 ng of control chicken DNA with increasing amounts of *T. cruzi* DNA, respectively: 1 fg, 10 fg, 1 pg, and 100 pg, and 1 ng. The hybridization with the radiolabeled 188-bp probe improved the technique sensitivity (10 fg), which reached 24-fold below the diploid *T. cruzi* total DNA.

To separate possible roles played by parasite persistence and autoimmunity in the pathogenesis of the inflammatory Chagas heart disease seen in *T. cruzi-*infected mammals, active infection coexisting with any mechanism of tissue inflammation had to be eliminated [19]. The variable of parasite persistence was removed by using a ‘clean’ host model [10, 42, and 53]. Thus, we used chickens refractory to *T. cruzi* infections and performed invasion studies early in their embryonic life. We inoculated 100 *T. cruzi* trypomastigotes into the air chamber of 60 stages X chicken eggs prior to incubation. The infection was established in the embryo cells (**Figure S1A to E**), and embryonic tissues collected on the second, fourth, and eighth days postinfection produced nDNA and kDNA amplifications; interestingly, tissue collected on the tenth, 12^th^, 18^th^ and 20^th^ days yielded amplification products only for kDNA (**Figure 1A**). To avoid the possibility of very low level of parasitism remaining in the embryo, we used a PCR assay with Tcz1/2 primers, and the amplicons obtained were subjected to hybridization with the radio labeled 188-bp nDNA probe to increase sensitivity of the technique [54], [55]. This assay can detect 10 fg of *T. cruzi* DNA (**Figure 1B**), which is 24-fold below the amount found in the diploid parasite [22]–[24].

**Figure 2:**
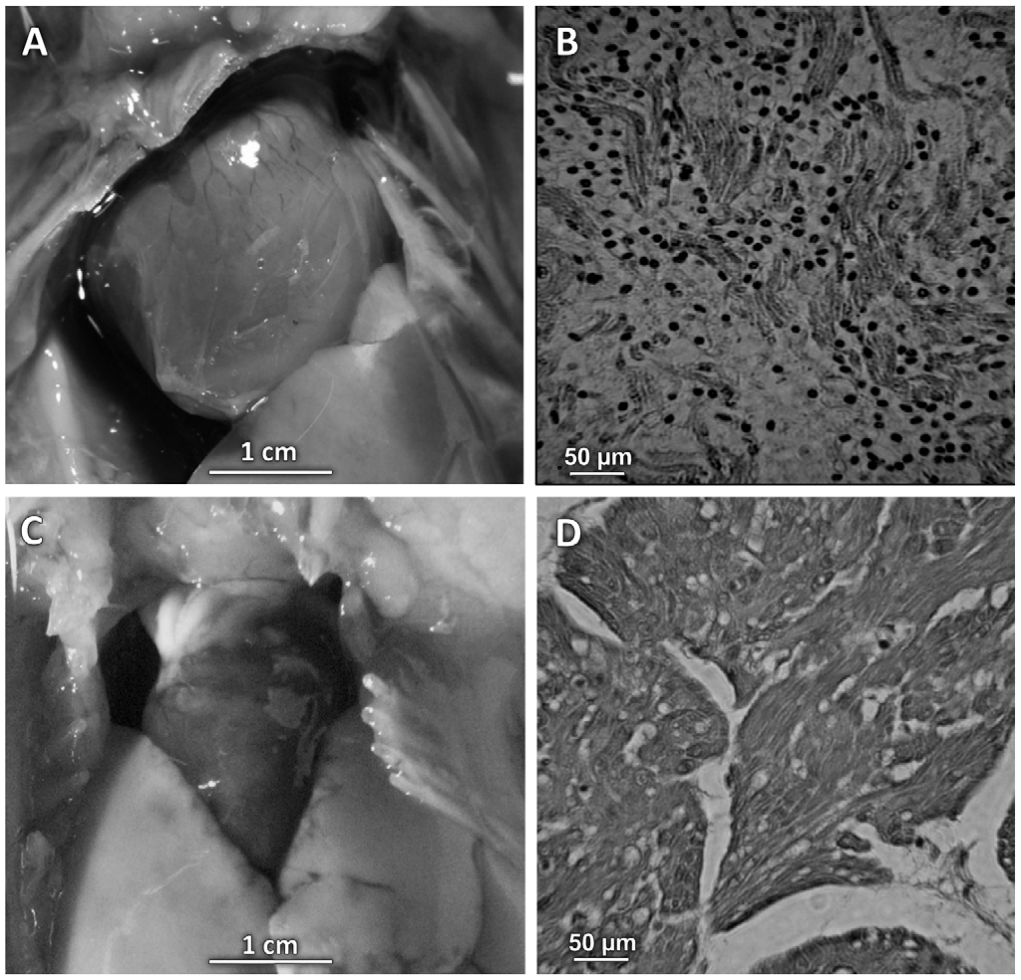
*Trypanosoma cruzi-*free inflammatory myocardiopathy in one week-old chick. A) Cardiomegaly in a chick hatched from *T. cruzi* inoculated egg. B) Histopathology showing severe inflammatory infiltrates and non-parasitized heart cell lyses by cytotoxic lymphocytes. C) Normal heart size of a mock control chick. D) Normal heart histology of control chick. H-E staining, magnification 200 X.

Among 48 *T. cruzi-*infected eggs that sustained embryo development, 28 (58.8%) hatched healthy chicks and 20 (41.2%) resulted in embryo liquefaction during the first week of growth (45%), and in deaths either at hatching (31%) or within one week after hatching (24%). The histopathology of whole body tissues from chicks that were found dead at hatching revealed severe inflammatory infiltrates and lyses of self tissues in the liver, kidneys, intestines, skin, lung, skeletal muscles and heart (not shown). Four chicks showing retarded growth and respiratory distress died during the first week of life. Those chicks had heart failure with cardiomegaly and inflammatory infiltrates with destruction of nonparasitized heart cells (**Figure 2**). None of these findings were present in 36 mock control eggs inoculated with 10 µl of culture medium in the air chamber, neither in 12 non-infected control eggs. In the control groups were found dead embryos (15.4%) in the first week of growth. The mortality ratios differences between groups of *T. cruzi* inoculated eggs and controls were highly significant (p<0.005).

Although the refractory nature of birds to *T. cruzi* is well known [10], [42], [53], we documented the absence of the active infection in each of 12 parental kDNA-positive (FO) chicks showing negative blood culture in axenic liver infusion-tryptose medium and negative blood inoculations in weaning mice. In the positive control tests, 50 µl aliquots from suspensions of blended tissues (50 mg/1 ml PBS, pH 7.4) from five day-old embryos, which had received *T. cruzi* in the air chamber, were inoculated in the peritoneal cavity of weaning mice and in axenic culture medium and yielded, respectively, blood trypomastigotes and epimastigote forms.

To further dissociate the kDNA retention event from the presence of active infection, we inoculated naked minicircle sequences in the air chamber of eleven embryonated chicken eggs. Absence of kDNA PCR products from these embryos tested weekly prior to hatching indicated that transfer of minicircle sequences to the chicken genome required a living *T. cruzi* infection in first week of embryonic growth. With this respect, further important information yet could be obtained in the crosskingdom model system, aiming at the documentation of the kDNA integration in the chicken genome, which were considered deemed necessary to clarify the pathogenesis of the parasite-free cardiomyopathy in chicks hatched from the *T. cruzi*-infected embryonated eggs.

### Retaintion of parasitic mitochondrial kDNA minicircle sequences

Having shown that the live *T. cruzi* infections were eradicated by the chick innate immune response after 10 days of embryonic development we fathomed the parasite mitochondrial kDNA alone that was shown in **Figure 1A**. The DNA templates from peripheral blood cells of F0, F1, F2 and F3 chickens (**Table S1**) were subjected to direct PCR amplifications, cloning and sequencing. A total of 25 kDNA minicircle sequences were obtained with 404±150 nts comprising conserved and variable minicircle sequence fragments (EMBL accession numbers: FR719694 to FR719718). In view or the reported hypervariability of the kDNA minicircles [30], [31] it was interesting to observe that 64% of these sequences retained in the chicken genome showed high similarity (e-values 5e-45 to zero) with those resulting from our investigations in humans [34]. This finding suggested that some classes of kDNA minicircles from *T. cruzi* may be preferentially retained in the vertebrate host, and that the minicircle sequences could be possibly integrated in the chicken genome [34], [37]–[39].

### Lateral kDNA transfer (LkDT)

**Figure 3:**
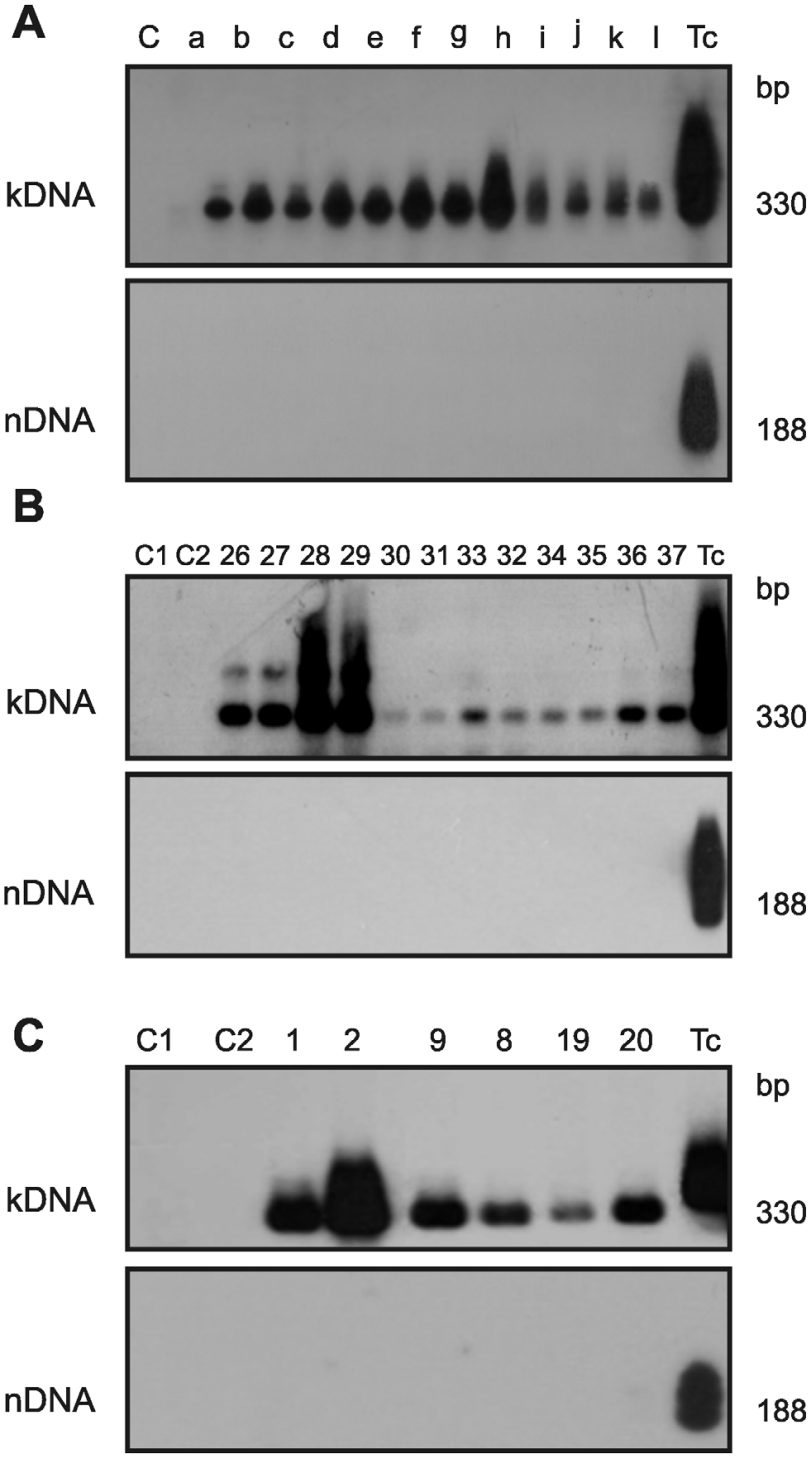
Retention of the kDNA minicircle from *Trypanosoma cruzi* in the *Gallus gallus* genome. A) PCR amplification of kDNA from 12 adult chickens hatched from *T. cruzi*-infected eggs with primer set s35/s36 and hybridization with *Nsi*I-digested, whole kDNA labeled as probe. The lower panel shows the absence of nDNA by the Tcz1/2 primer set, hybridized with a 188 bp probe. B) *G. gallus* somatic cell's DNA templates from F3 progeny, which were separated in 1% agarose gels, revealed the minicircle 330-bp band in the absence of nDNA by using specific primer sets and hybridization with the 188 bp probe. C) *G. gallus* germ line DNA templates from parental 1 and 2, and from mated progeny in the F1 (8 and 9) and F2 (19 and 20) amplified with primer sets s35/s36 or Tcz1/2 and probed as described.

The documentation of parasite-free inflammatory cardiomyopathy (**Figure 2**) in chicks hatched from eggs that had received the *T. cruzi* inoculations incited us to continue the investigation about kDNA integrations and resulting genotype alterations in the chicken model system. DNA templates were obtained from twelve chickens hatched from infected embryos, which showed *T. cruzi*-kDNA amplicons in the absence of parasite nDNA (**Figure 3A**). In the *T. cruzi*-free control experiment 12 embryonated chicken eggs and 36 mocks were subjected to PCR, and neither nDNA nor kDNA was detected. Therefore, it was clear that kDNA alone was transferred to the chick genome during the transient *T. cruzi* embryonic infections. The horizontal transfer of kDNA minicircle sequences to parental (F0) chicken genomes could have physiopathological consequences that would be valuable in a model to study the pathogenesis of chagasic heart disease. Therefore, F0 birds were raised for crossbreeding. The F1, F2, and F3 progeny tested positive for the kDNA in lack of nDNA, indicating that the *T. cruzi* infection occurring early in the embryonic developmental process generated mature chicken with kDNA integrated into gonadal tissue (**Figure 3B**).

### Vertical kDNA transfer (VkDT)

Sperm and ova from birds hatched from *T. cruzi*-infected eggs was examined because it was fundamental to confirm vertical transfer of minicircle sequences to progeny via the germ line. DNA templates of germ line cells from roosters and hens yielded PCR amplicons with kDNA s35/s36 primers but lacked nDNA amplification with the Tcz1/2 primers (**Figure 3C**). Crossings of kDNA-mutated F0 birds generated F1, F2 and F3 progeny and each sibling showed amplicons of minicircle alone. A pedigree depicting LkDT into parentals and VkDT into chicken's progeny is shown in **Figure S2**.

In control experiments template DNAs were subjected to PCR, and neither nDNA nor kDNA was detected. Southern blot analyses of *Eco*RI and of *Mbo*I digests of DNA from body tissues (blood mononuclear cells, heart, skeletal muscle, liver and kidney) of parental F0, and offspring F1 and F2 progeny revealed various size bands with a kDNA-specific probe (**Figure S3A and B**). The various positions occupied by the kDNA bands in Southern blots revealed that minicircle sequences were integrated in the parental and offspring chicken genomes.

Thus, chickens with kDNA integrated into their germ line and somatic cells in the absence of the infection were generated. The detection of kDNA signals on fragments of distinct sizes from unintegrated minicircles in the heart DNA of chickens combined with the absence of *T. cruzi* nDNA attests to the success of the integration event and of the subsequent eradication of the infection.

### Mapping *Trypanosoma cruzi* minicircle integrations in the *Gallus gallus* genome

**Figure 4:**
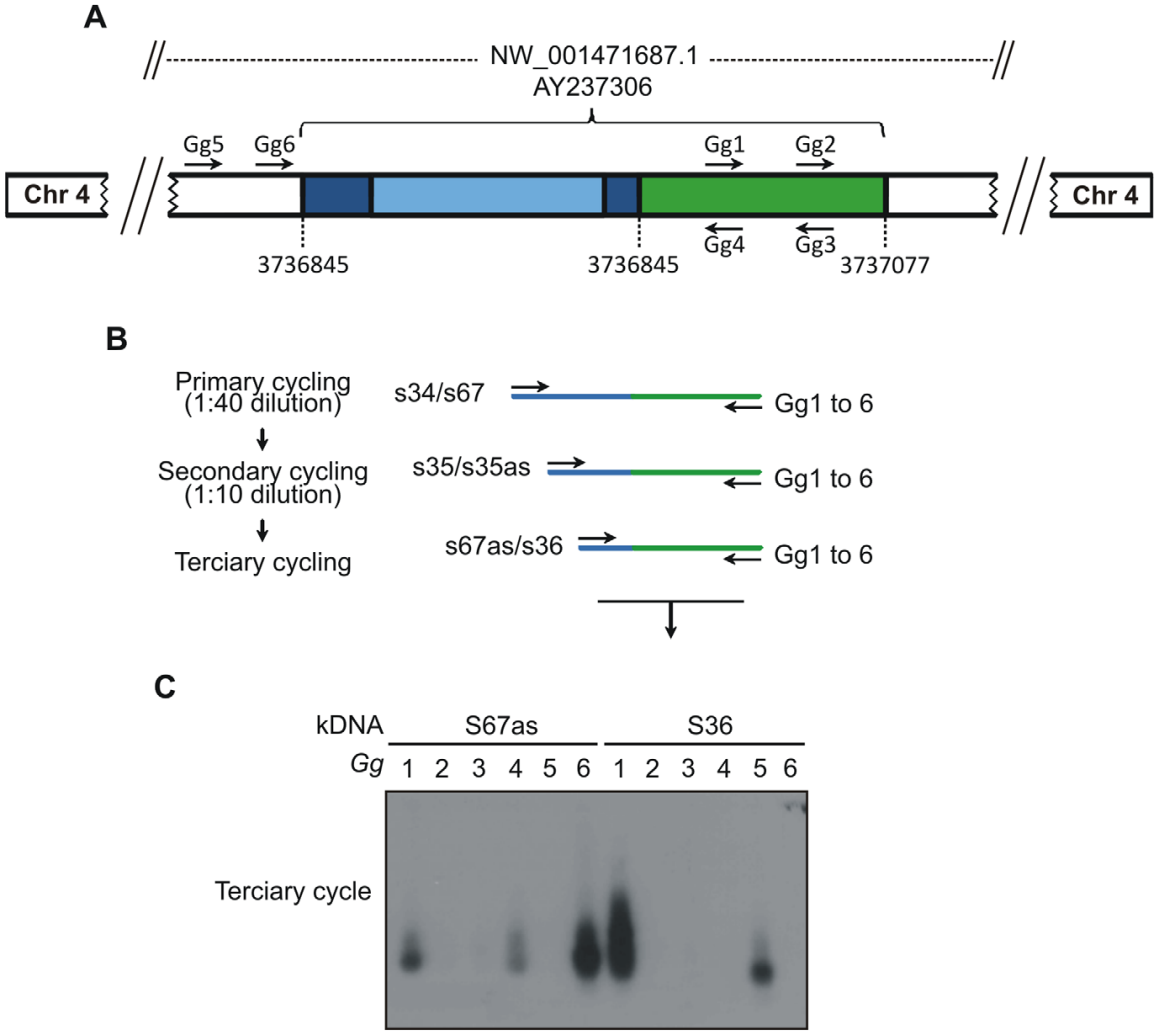
The *tp*TAIL-PCR strategy used to detect *Trypanosoma cruzi* kDNA integration into the *Gallus gallus* genome. A) A chimera sequence with a fragment of kDNA minicircle conserved (dark blue) and variable (light blue) regions integrated in the locus NW_001471687.1 at chromosome 4 (AY237306) of the chicken genome (green) was used to obtain the host specific primer sets (*Gg*1 to *Gg*6). B) The *tp*TAIL-PCR amplifications were initiated (primary cycle) by annealing of the kDNA-specific S34 or S67 primers in combination with chicken-specific *Gg*1 to *Gg* 6 primers. Diluted products provided template for the secondary cycle with the S35 (sense/antisense) primers and the combinations of *Gg* primers. In the tertiary cycle a dilution of the secondary products was subjected to amplification with kDNA S36 or S67 antisense primers in combination with the *Gg* primers. C) These amplification products were separated in 1% agarose gels and transferred to nylon membrane, hybridized with the specific kDNA probe, then cloned and sequenced. The combinations of kDNA and targeted *Gg1* to *Gg6* are shown on top of the gel. The sequential PCR reactions amplified target kDNA-host DNA sequences with kDNA minicircles (blue) and the avian sequence (green).

The difficulty in demonstrating randomly contemporaneous eukaryotic interspecies DNA transfer may be explained partially by the inaccessibility of those events to an effective methodological approach. We obtained by chance a chimeric kDNA-chicken DNA sequence (AY237306), which was amplified by the 5′-RACE technique. This model sequence was used to construct primer sets *Gg1* to *Gg6* **(** **Table 1**) annealing upstream and downstream to a minicircle integration event into the locus NW_001471687.1. The substitution of traditional PCR degenerate primers by host DNA-specific primer sets *Gg1* to *Gg*6 eliminated the main difficulty and permitted demonstration of the junctions of kDNA-host DNA chimeras, employing a targeted-primer thermal asymmetric interlaced-PCR (*tp*TAIL-PCR). A scheme with the strategy used to amplify the kDNA integration event in the *G. gallus* genome is shown in **Figure 4**. The description of the modified *tp*TAIL-PCR is given in the Methods section, and a detailed flowchart with primer set combinations used is shown in **Figure S4A to C**. The *tp*TAIL-PCR was then employed to amplify minicircle-host DNA junction sequences from our chickens. The amplicons that tested positive with a radioactive kDNA probe were cloned and sequenced (FN598971 to FN590000, FN599618, FN600557, and FR681733). These results are shown in **Table S1**.

In control experiments the *tp*TAIL-PCR products did not test positive with the specific kDNA probe. Validation experiments consisted of *tp*TAIL-PCR amplifications of a mix of *T. cruzi* kDNA with control chicken DNA (**Figure S4A to C**). Twenty-three amplicons that tested positive with the wild-type kDNA probe showed only kDNA sequences with no host contribution.

**Figure 5:**
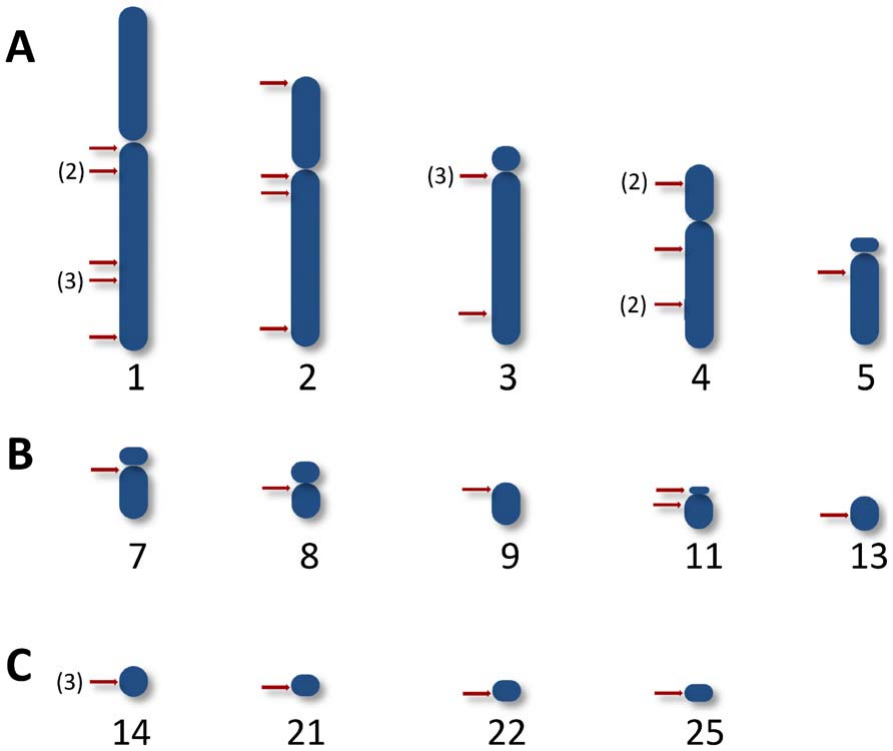
Heredity of the integrations of *Trypanosoma cruzi* kDNA minicircles into several loci of the chicken genome. Rows A, B, and C show the integrations, respectively, in the macrochromosomes, in the intermediate, and in the microchromosomes. The numeral(s) in brackets indicates the total times an insertion (red bar) was present at a chromosomal locus from animal source shown in Table S1.

Chimeric sequences with minicircle-host DNA junctions were obtained from chickens testing positive by PCR with the minicircle-specific s35/s36 primers. Thirty-four chimeras (total F0, 8; F1, 17; F2, 7; and, F3, 2) with average 555±153 nts (kDNA 296±78 nts and host DNA 281±148 nts) were documented in 14 chromosomes. *E*-values for each of the chimeras were statistically significant (p<0.001; kDNA, 1e^−06^ to 2e^−150^; host DNA, 1e^−53^ to 0). Three of these chimeras were obtained by using chicken repeat-1 (CR-1) specific primers (FN598975, FN598994, and FN598998). The minicircles spread to various loci of chicken chromosomes are shown in **Table S1**. Overall, 64.6% of kDNA-mutations entered in the macrochromosomes (1, 38%; 2, 18%; 3 18%; 4, 23%; and, 5, 3%), 17.7% in the intermediate, and 17.7% in the microchromosomes of the chicken genome. A map showing the heredity of the kDNA integrations in those chromosomes loci is depicted in **Figure 5**. BLASTn analyses revealed that *Gg*1 to *Gg*6 primer sets aligned to multiple loci in 18 chicken chromosomes with the following frequencies: *Gg*1, 19; *Gg*2, 39; *Gg*3, 28; *Gg*4, 19; *Gg*5, 3; and, *Gg*6, 23. Thus the *tp*TAIL-PCR achieved reproducible random amplification of kDNA-host DNA integrations in a variety of chromosomes. The alignment of chimeric sequences from F0 (AY237306) and F1 (FN600557) chickens documented vertical transfer of the kDNA mutation in non-coding locus NW_001471687.1 of chromosome 4 **(Figure S5A)**. In addition, kDNA mutations in the dystrophin gene locus NW_001471534.1 at chromosome 1 from F1 (FN598991) and F2 (FR681733) progeny showed perfect alignments (**Figure S5B**). The heritability of the kDNA mutations was documented; the fixation of the mutations in the chicken model can be further spanned through obtaining host's specific primer sets anneal to the kDNA-mutated *loci* and the full sequencing of the targeted chromosome.

### End-joining microhomologous recombination intermediates kDNA integration

Due to the CA-rich microhomologies in chimeric sequences detected in the genomes of Chagas disease patients [34]; we conducted a bioinformatic search for similar features in the kDNA-mutated chick sequences, revealing CArsb repeats between kDNA-host DNA junctions. Sequence analyses of CArsb repeats intermediate to the kDNA minicircle integration into the chicken genome revealed consensus I – ACACCAACCCCAATCGAACCCAAACCAAA, present in seventeen clones, and consensus II – TAYACCMACCCCTCCCAAAACC, found in the flanking region of eleven chimeras. CArsb microhomologies in chimeric sequences secured from kDNA-mutated chickens are depicted in **Figure S6A and B**. These repeats found coding regions in the chicken genome concentrated (52.4%) in chromosomes 4 (13. 5%), 3 (5%), 2 (20.4%) and 1 (13.5%). Additionally, CArsbs were also present in long terminal repeat Hitchcock transposons (FN598974 and 599618), and in CR-1 non-LTR retrotransposons (FN598975, 598994, and 598998), and L1-24xT (FN598995). The consensus microhomologies in coding regions, chicken LTRs and non-LTRs, and minicircles implied that microhomology-mediated end-joining [56] was mediating integration of exogenous sequence into host chromosomes.

### Minicircle disruption of host genes

The data shown in **Table S1** were obtained from DNA templates of F0, F1, F2 and F3 chickens with inflammatory cardiomyopathy. A range of minicircle integration events promoting rupture of ORFs of those chickens is shown in **Table S2**. Twenty kDNA integrations (∼60%) were detected in coding regions of various chromosomes. These integrations were seen frequently in genes encoding protein kinases (20%) playing important roles in cell division and differentiation, in the dystrophin gene (10%), which encodes a high molecular weight protein connecting the cytoskeleton to muscle and nervous cell membrane, and in growth factors (10%), transcription factors (5%), and immune factors (5%). Other important genes encoding GTPase, adenylate cyclase, and adhesion molecules related to macrophage recruitment and blood vessel maturation were disrupted. In one case a gene expressed in blood mononuclear cells from patients with systemic lupus erythematosus (NW_001471554.1) was ruptured by kDNA integration (FN598994). A minimum of 12 mutations were observed in one chicken with severe inflammatory cardiomyopathy. These mutations may skew coding regions of chromosomes with subsequent functional alterations such as cell cycle regulation, clonal proliferation of immune system cells, and tissue injury [57]–[61]. Interestingly, documented clinic and pathologic manifestations were clearly associated with the kDNA-mutations in the locus of the dystrophin gene (**Figure S5**) in two chickens with muscle weakness, cardiomegaly and heart failure.

### Identification of ORFs in chimerical sequences

The chimerical sequences that were obtained from F0, F1, F2, and F3 kDNA-mutated chickens (**Table S3**) presented ORFS with the potential for translation of hybrid proteins. A total of 13 ORFS (43.3%) comprised kDNA alone, and 17 ORFs (56.7%) were chimeras formed by kDNA-host DNA. A majority of the ORFs (77%) encoded proteins without significant similarity, but 20% of the ORFs translated hypothetical proteins with significant similarities (e-values ranging from 2e-06 to 2e-22) with other proteins. Furthermore, one ORF encoding the reverse transcriptase from *G. gallus* (*locus* AA49027.1) showed highly significant scores (4e-29). In the model system used each ORF encoding putative neo-antigen was generated after the invasive *T. cruzi* replicated in the embryonic tissues prior to the development of the chick immune system in the first week of growth. Therefore, a functional role for ORF's encoded neo-antigen in the pathogenesis of Chagas disease did not hold promise in the absence of humoral autoimmune factors in the actively tolerized kDNA-mutated chicken [62]–[66].

### Identification of gRNAs in the kDNA minicircles

**Figure 6:**
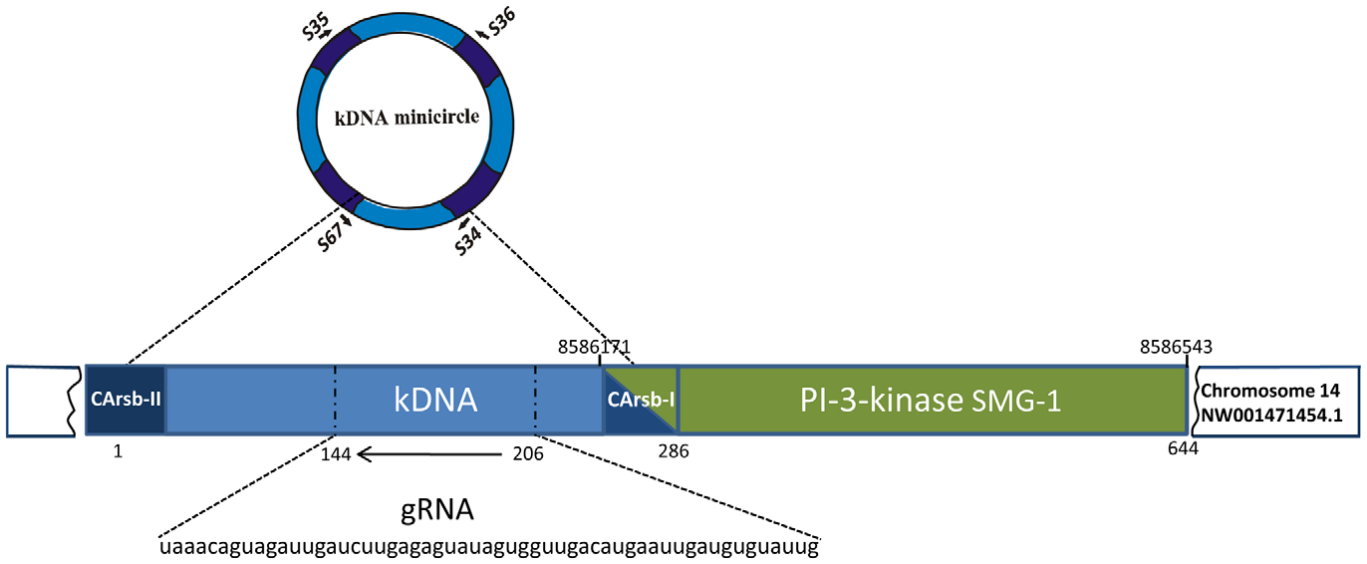
Representation of the gRNA in the *Trypanosoma cruzi* kDNA minicircle integrated into *Gallus gallus* genome. The kDNA conserved (dark blue) and variable (light blue) fragment (nts 1 to 286) is inserted in the PI-3K serine-threonine related kinase SMG1 (Supressor Morphogenetic Genitalia) at the locus NW_ 001471454.1. A CA-rich sequence block (CArsbI) microhomology intermediates the kDNA integration into the PI-3K exon. A gRNA cognate to Tbnd7ed (**Table 2**) is present in the kDNA variable region (dotted line), which is formed by 55 nts in antisense direction (arrow). Short arrows indicate positions of kDNA primers.

The chimerical sequences showing kDNA integrated into the host chicken chromosomes presented hypervariable minicircle regions (**Table S1**) with the potential for gRNA transcription [29]–[31], [33], [35], [36]. The analysis of these hypervariable sequences determined significant similarities with those edited maxicircle gene sequences in KISS database, using the WU-Blastn-modified-matrix [52]. This approach allowed the G-U base pairing and retrieval of sequences with highly significant alignments. In total the approach revealed putative gRNAs in six out of the 34 chimeras kDNA-host DNA (**Table 2**). The sequences showing best alignment scores (e-values 7.9e- 3 to 9.3e-06) showed cognate gRNAs adequately positioned in the hypervariable region of the minicircles (**Figure 6**). Consistently, the gRNAs (54±6 nts) were located 56±5 bp from the CArsb-I. Furthermore, the predicted aminoacid similarities of 96.1, 89.4 and 95.2%, respectively, for the NADH dehydrogenase subunit 7 (NAD7), ATPase 6, and ND8 edited *T. brucei* and *T. cruzi* matched genes held high confidence to the identification of gRNAs in the integrated kDNA minicircles. The functional consequence of the parasite-derived gRNA editing minicircles in the vertebrate host is presently unknown.

**Table 2:** Identification of guide RNAs in the *Trypanosoma cruzi* mitochondrial kDNA minicircles integrated into the chicken genome.

Clone	Sequence producing significant alignment (Kiss Database)	*E-value* (Kiss)	BLAST hit	gRNA size (nt)	Distance (bp) from CArsb II*	Distance (bp) from CArsb I*	Sequence (5′ to 3′)
FN598973	Tbnd7ed	0.0055	gb|M55645.1|TRBKPDHAB Trypanosoma brucei kinetoplast NADH dehydrogenase subunit 7 mRNA, complete cds	43	146	57	UGUGUACGAUGACUAUGAUGUGAGUUGGAGAUUAUUAUUGACU
FN598974	Tba6ed	1e-05	gb|M33228.1|TRBKPEATA Trypanosomabrucei kinetoplast ATPase 6 edited mRNA, complete cds	56	153	57	AUAGAUGUGAGUUCAAGUAGGUAAUUCAGUGGUGUAAGAUUAGAUUGUGUAUAUUA
FN598978	Tbrps12ed	0.0034	gb|M77751.1|TRBCR6MC Trypanosoma brucei ORF1 and ORF2 (CR6) mRNA, putative cds	59	144	62	AUAGUAUGACAAUGAAGAUAACGUACAUGCUAGGUGAGGUUAUAUGAGUAAGAUACAGU
FN598982	Tbnd7ed	0.0079	gb|M55645.1|TRBKPDHAB Trypanosoma. bruceii kinetoplast NADH dehydrogenase subunit 7 mRNA, complete cds	55	141	59	UAAACAGUAGAUUGAUCUUGAGAGUAUAGUGGUUGACAUGAAUUGAUGUGUAUUG
FN598984	Tbrps12ed	9.3e-06	gb|M77751.1|TRBCR6MC Trypanosoma brucei ORF1 and ORF2 (CR6) mRNA, putative cds	61	115	56	UUAUAGAGUGAUGUAAGACAAAUGAGAUAGAACGUGGAUAAUAGUUGUGUGAAGAUUAUAU
FN598997	TO270442_ND8BSA12M13R_B23.ab1	0.004	gb|M63820.1|TBNADHDS Trypanosoma brucei kinetoplast putative NADH dehydrogenase subunit 8 (ND8) mRNA, complete cds	51	153	48	GAUGUAUAUAAAGAUGGACAAGAGAAGAGAUGAAACGUUGAGAGAAAAUGU

*CArsbs I and II, CA-rich minicircle sequence blocks equivalent to constant sequence blocks (CSBs) 3 and 1 [32] are depicted in Figure 6.

### Clinic manifestation of heart disease in kDNA-mutated chickens

**Figure 7:**
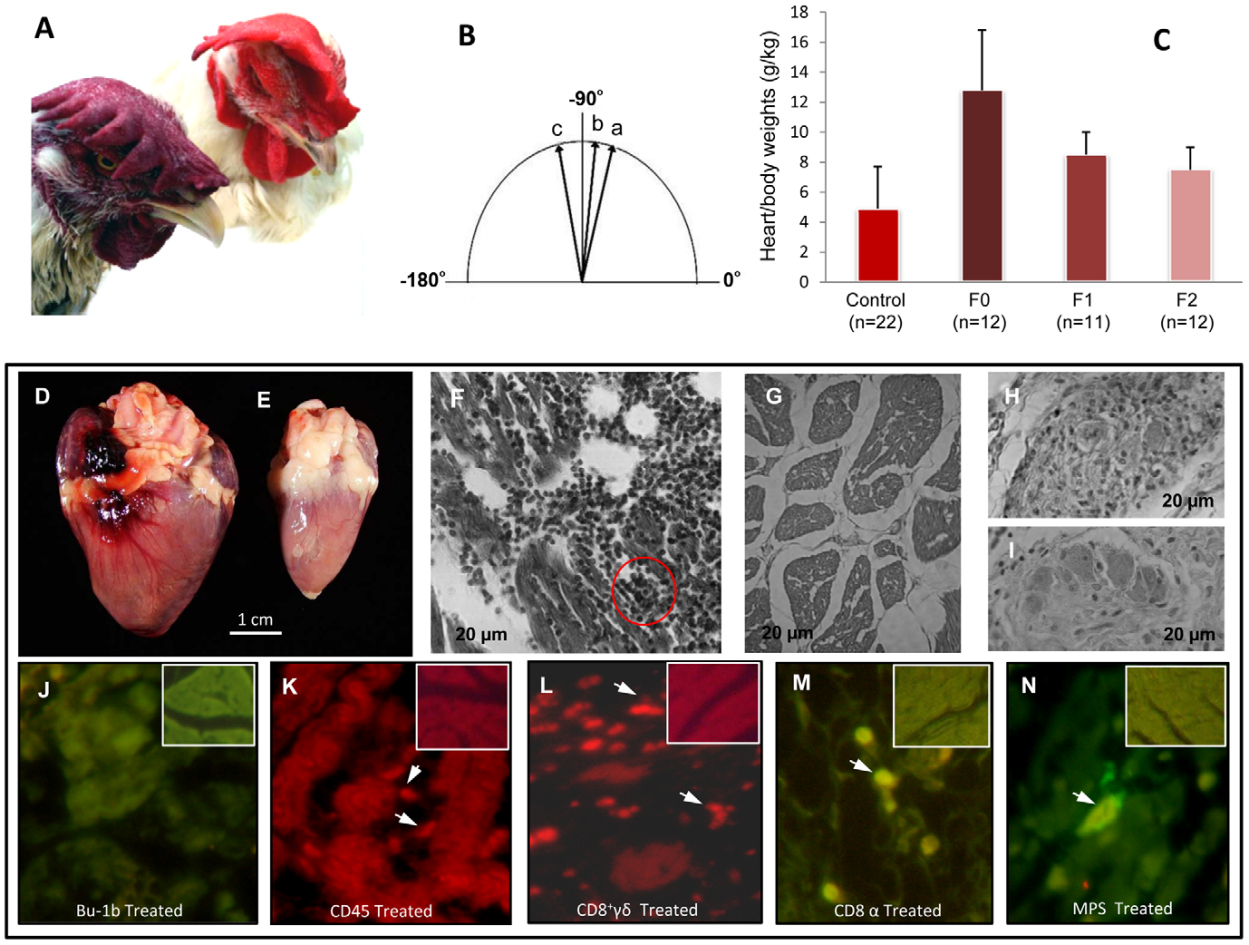
Clinical and pathological findings in *Gallus gallus* with *Trypanosoma cruzi* kDNA mutations. **A**) Nine month-old F1 hen displaying heart insufficiency by cyanosis of the comb (bottom left), and a control hen of the same age showing a bright red comb (top right). **B**) Deviation of cardiac axis from *a*-to-*c* over a six-month period. **C**) Increased cardiac heart/body indexes in chickens with kDNA integrations. The heart/body size indexes showed statistically significant differences (*p≤*0.05) in control and in kDNA-mutated chickens. D) Cardiomegaly (30 g) in a nine month-old hen that died of heart failure. E) Control heart (8 g) from a nine month-old hen. F to I, H-E, magnification 100X: F) Diffuse myocarditis showing immune system mononuclear cell infiltrates and lysis of target heart cells. The red circle depicts a minimal rejection unit whereby effectors lymphocytes destroy a target heart cell. G) Histology of control chicken heart. H) Intracardiac parasympathetic ganglion showing mononuclear cell infiltrates and neuronal cell lysis. **I**) Control plate showing normal histology of an intracardiac parasympathetic ganglion. J to N, series of histological analyses with kDNA-mutated chicken heart; control uninfected chicken heart tissue shown in the inserts: J) Lack of B cells in a destructive heart lesion treated with anti-Bu-1 monoclonal antibody. K) CD45^+^ lymphocytes identified (arrows) in heart lesions by a phycoerythrin-labeled specific monoclonal antibody. L) CD8^+^γδ immune lymphocytes (arrows) involved in severe destruction of the heart. M) Abundant CD8α^+^ T cells present in severe lesions with heart cell lysis. **N**) Mononuclear peripheral cells, monocytes and macrophages in the heart lesions.

We inspected the kDNA-mutated and as well control chickens daily for mortality and weekly for clinic manifestations of disease. Often, the kDNA-mutated chickens showed signs of shortness of breath and impaired oxygenation of blood that evolved to severe cyanosis (**Figure 7A**). The electrocardiograms recorded at three and six months of age (**Figure 7B**) in 12 F0 kDNA-positive birds and in 22 control chickens never exposed to *T. cruzi* showed that controls retained the electric axis at +75° and test birds changed axis positions to the left from +80 to –115° over time. The heart/body weight indexes from kDNA-positive F2, F1 and F0 birds ranged, respectively, from 6±2, to 6.7±2, and to 12±5, whereas the control group index was maintained a constant 4.2±2 (**Figure 7C**). The differences among high heart indexes from F0 and from F1 kDNA-mutated chickens are statistically significant from the control low indexes (p<0.05). Survival lengths for the F0 and F1 kDNA-positive birds were shorter for F0 (12±4 months) and F1 (13±2 months) than those in the control group (19±5 months), and these differences were statistically significant (p<0.05).

### Pathology and pathogenesis

Cardiomegaly was documented in 65% of the kDNA-mutated adult birds, and absent in control animals free of minicircle sequences. In the case of F1 hen 9 (**Figure 7D**) the heart weight was over three times that of a control bird of same gender and age (**Figure 7E**). Pleural and peritoneal effusions were collected in kDNA-positive birds with cardiomegaly and heart failure. The microscopic examinations of sections from the myocardium showed severe infiltrates of immune system effector lymphocytes and target cell lyses (**Figure 7F**). This destruction of parasite-free target fiber by effector cells was typical, characterizing a minimal rejection unity in the hearts of kDNA-mutated chickens (red circle). These microscopic features were absent from control chicken hearts (**Figure 7G**). Furthermore, the coalescence of several rejection units resulted in diffuse myocarditis with massive destruction of the myocardium in chickens showing cardiomegaly. The intracardiac parasympathetic ganglion also showed mononuclear cell infiltrates and destruction of neurons (**Figure H**). These pathologic features were neither encountered in intracardiac ganglia nor in myocardial sections from controls (**Figure 7I**).

The phenotype of immune system mononuclear cell infiltrates in sections of myocardium from kDNA-positive birds revealed a lack of Bu-1b treated B-cells associated with humoral immune responses (**Figure 7J**). By contrast, sections of myocardium of kDNA-positive birds treated with anti-CD45, anti-CD8γδ, or anti-CD8α showed specific staining of immune lymphocytes that carry out lysis of target heart cells (**Figure 7K, L and M**). Treatment of those sections with specific antibodies revealed that some cells in the myocardium infiltrate bore the macrophage phenotype (**Figure 7N**). In control experiments, sections from myocardium of control birds (white-frame inserts) showed neither markers of immune system cells nor tissue destruction. A possible role played by Th17 and Treg immune responses [67]–[71] in the destructive myocardial lesions requires investigations in the chicken model.

Actually, the typical inflammatory type autoimmune myocarditis depicted in F0 and F1 chickens is also observed in F2 progeny, albeit to a much lesser frequency. A F2 chicken that showed cardiomegaly and succumbed to heart failure (**Figure S7**) had the kDNA mutation in an exon of the dystrophin gene (FR681733). The inflammatory cardiomyopathy with lymphocyte rejection of target heart cells, typical of the autoimmune Chagas-like disease in the kDNA-mutated chicken model system, was attenuated in the F3 generation, which reached the adult life two years after hatching without clinical signs of a heart disease. Accordingly, the kDNA mutations were ranked in four levels: a) High letality and early embryonic death; b) Age group specific heart disease; c) Neutral in lack of disease manifestation; d) Possible beneficial, yet difficult to demonstrate. In this regard, attenuation of a kDNA mutation was defined by the decreasing levels of manifestations encountered in the chicken model system.

## Discussion

To separate the roles that parasite persistence and autoimmune rejection of target tissues play in the pathogenesis of the Chagas heart disease, implementation of an animal model that does not retain cryptic *T. cruzi* infections was essential [10]. In this respect, the mature chicken immune system is considered a tight biological barrier against *T. cruzi*. In this study we describe a *G. gallus* model that fulfills the criterion: *T. cruzi* infection is eradicated by the innate immunity present in the chicken embryo upon development of its immune system by the end of the first week of growth [10]. Here we demonstrate that chicks hatching from *T. cruzi-*inoculated eggs eliminate the live infection, lacking the parasite nDNA. Additionally, these chicks retain *T. cruzi* minicircle sequences in their genome, and these mutations are transferred to their progeny. The kDNA mutations integrated in coding regions of multiple chromosomes. The integrations ruptured open reading frames for transcription and immune system factors, phosphatase (GTPase), adenylate cyclase and phosphorylases (PKC, NF-Kappa B activator, PI-3K) associated with cell physiology, growth, and differentiation [57]–[59]. Severe myocarditis due to rejection of target heart fibers by effector cytotoxic lymphocytes is seen in the F0 and F1 of the kDNA-mutated chickens, showing an inflammatory cardiomyopathy similar to that seen in Chagas disease. Interestingly, heart failure and skeletal muscle weakness were directly associated with the kDNA mutations and rupture of the dystrophin gene in chromosome 1 of adult chickens [72], [73]. Moreover, the contribution of various mutations present at other loci in the genomes should be emphasized, because those chickens with kDNA integrations spread throughout their chromosomes also presented the self-tissue destructive pathology. Cardiomegaly and heart failure recorded for F0 and F1 kDNA-positive birds consistently attenuate in F2 and F3 progeny. Thus kDNA-integrations in some chromosome coding regions, generating skewing, instability, and clonality [60], [61], [74], may undergo long-range intragenomic signaling interactions [75], so as to achieve physiological balance over forthcoming generations of descendents; in our experimental system, however, the absence of active *T. cruzi* infection is clear.

Experimental *T. cruzi* inoculation of the chicken embryo highlights the crosskingdom exclusion of infection that prevents evolutionary consequences resulting from the lateral transfer of parasite DNA to the bird genome. We document these conditions to confirm that only a narrow window is open for the infection to become established within the first week of a chicken embryonic life. In the absence of a mature immune system barrier, early intracellular multiplication of *T. cruzi* in embryo stem cells is possible. Inoculation was performed at the epiblast stage of chick development at which all embryonic cells are susceptible to *T. cruzi* invasion, and the kDNA can integrate in stem cells that differentiate both somatic and genital crest precursors of germ line. If this phenomenon were possible in nature it would create the opportunity for increasing genetic diversity and evolution of the species undergoing continuous change on a grand scale over time [34]. In this regard, four categories of functional kDNA mutations are described in this study: The high letality mutations that generate abortions, congenital inflammatory cardiomyopathy, and early death, in which the genotype modifications by means of DNA transfer result in pathology incompatible with life (negative selection) [10]. Age group specific mutations may be attenuated in a majority of chickens that succumbed to the Chagas-like inflammatory cardiomyopathy late in adult life. Neutral kDNA-mutations are probably present in 35% of the chickens not compromised by heart disease; these neutral mutations may contribute to genome growth and positive selection. Theoretically, beneficial mutations may exist [76], but they could not be identified in three generations of kDNA mutated chickens.

The autoimmunity in Chagas disease was proposed to explain about a preformed capacity of immune lymphocytes to carry out an accelerated destruction of non-parasitized target heart cells within few hours of incubation [16], and, thereafter, it was suggested that existing cross-reactive antigens in target tissues would call in the *T. cruzi-*sensitized lymphocyte cytotoxicity [77]–[79]. However, the attempts to reproduce the myocarditis by immunization of laboratory animals with parasite recombinant antigens resulted in small infiltrates of mononuclear cells in absence of clinical symptons and of gross lesions [80]–[83]. The molecular mimicry mechanism was suggested to explain the autoimmunity, whereby cross-reaction of parasite antigen-immune effector cell against self-antigen on target cell, sharing putative similar amino acid motifs or three dimensional epitopes, was required to trigger off self-tissue rejection [84]–[92]. Accordingly, mimicring immunogenic cryptic self peptides may become accessible to auto-reactive T-lymphocytes that escape from the host's central and peripheral tolerance mechanisms [93], [94]. In this regard, molecular mimicry between cardiac myosin heavy chain (residues 1442–1447 AAALDK) and *T. cruzi* protein B13 (residues AAAGDK) could generate autoimmunity [85]–[87], but it was shown that anti-myosin autoimmune factors was not essential for cardiac damage [93]–[95]. So far, gross and microscopic pathology, and clinic manifestations of Chagas disease have not been obtained yet, by traditional immunizations with wild or recombinant *T. cruzi* antigens and, therefore, the primary cause of autoimmunity in Chagas disease was not explained. In this study, we suggest that the pathogenesis of Chagas disease is genetically driven.

Herein, kDNA-mutated adult chickens are shown to develop gross cardiomegaly in association with clinic manifestations similar to those described for the human disease [10, 12, and 96]. The lethal cardiomyopathy in the parasite-free chicken model system in which the destruction of heart cells by lymphocytes is documented is used to validate the autoimmune pathogenesis of human Chagas disease. These phenomena were never seen in mock or control chickens. Interestingly, the chicks that die after hatching show cardiomegaly and myocarditis, with heart cell destruction by lymphocytes similar to that described for congenital human Chagas disease. Moreover, the inflammatory cardiomyopathy that is the hallmark of human disease was present in a significant portion of the *T. cruzi* kDNA mutated adult chickens and their progeny. In those chickens, the intensity of the self destructive inflammatory process varied from one region to another in the myocardium; while some lesions are triggered high, others are intermediary or in a feeble state. Thus, some areas in the heart may be spared while others may be affected harshly by the inflammation; the intensity of the process never reaches all the heart simultaneously, which would not be compatible with prolonged survival. Such features are evidence of a genetically-driven autoimmunity with the following progression: *i*) accumulation of minicircles integrated in germline and somatic cells; *ii)* rupture of important genes, such as those regulating cell growth and differention, and the immune responses; *iii*) heart damage produced by lymphocytic infiltrates and lyses of target cells; *iv*) age-group specific rates of the disease.

The Chagas-like disease in the chicken shows multifaceted clinical presentations involving primarily the heart and skeletal muscles, along with the peripheral nervous systems, leading to ominous repercussions in the cardiovascular system. These manifestations can be explained only by the *T. cruzi* minicircle sequence integrations at several loci in the genome. The tolerance mechanism in kDNA-mutated chickens could not discriminate between self and non-self target tissues because the immune surveillance, fundamental to keep the self constituents free of the destructive reactions from the body self-defense apparatus, may be dampened due to the genotype modifications. The anti-self lymphocyte destruction of the heart happens when breakdown of self-tolerance or deregulation of the surveillance mechanism occurs [62]–[66]. Thus, cardiomegaly with lymphocyte destruction of heart cells in a genotypically modified crosskingdom parasite-free model of the human Chagas disease is shown here for the first time.

Experimental *T. cruzi* infections of laboratory animals and natural infections of hundreds of mammal species and of man reveal a high prevalence of Chagas heart disease and no cancer [1], [8]. This study shows that the key environmental factor contributing to the development of autoimmunity and self-heart destruction in the chicken model and in the human Chagas disease is the *T. cruzi* infection, during which the transfer of the kDNA minicircle to the host's genome occurs. Within this context, the host's immune system interacts in the conventional way to afford partial protection against *T. cruzi* infection, whereas autoimmune disease may ensue from genotypically modified T-cells producing clonal cytotoxicity. An intrinsic feature of the minicircle sequences in the kDNA mutations may produce genotype alterations with rupture of genes regulating cell growth and differenciation factors, but not cancer. We suggest that the generation of autoimmune disease in mammals and in birds might be an intrinsic feature stemming from the protozoan kDNA. This hypothesis requires further investigations.

The typical inflammatory cardiomyopathy is present only in chickens with somatic mutations hatched from *T. cruzi*-infected eggs. These kDNA-mutated chickens show early mortality in comparison with controls. When these chickens die the conspicuous pathologic finding is the inflammatory infiltrates with destruction of heart myofibers by immune system cytotoxic T-lymphocytes. The phenotype of immune system cells in the heart inflammatory infiltrates reveals that a thymus-dependent immune response, destroying the target tissue is a hallmark for pathogenesis of Chagas-like heart disease in kDNA-mutated chickens. In this respect, each kDNA-integrated immune system mononuclear cell involved in the ‘self’ tissue destruction is essentially a mutated clone [97], promoting an inflammatory lesion in the chicken heart. Each clone not withstanding thymic selection is considered an autoreactive T-lymphocyte repertoire producing the heart lesion, which is an important risk factor for disease outcome [98], [99].

The scattered nature of the minicircle integrations in CA-rich sites throughout the chicken genome indicates that a large number of host loci are susceptible to kDNA mutagenesis [32], [34], [40]. To determine the full extent of this phenomenon, complete sequencing of a kDNA-mutated chicken is required to identify the full cohort of minicircle integrations resulting from a single, specific infection event; each new introduction of the parasite will give rise to a unique combination of integrations for a given individual, resulting in a spectrum of clinical consequences. Although we have documented the disruption of multiple chicken genes resulting in compromised immune system self-tolerance that became permissive to autoimmune rejection of target tissue, inflammatory cardiomyopathy and failure, several integration events may be associated with these phenotypes. Accordingly, 20 genes and five X-linked disorders correlate with manifestations of failure in the genetic etiology of the heterogeneous group of cardiomyopathy in humans [98]–[102]. Thus, groups of integration mutations, and combinations thereof, may explain the clinical symptoms associated with Chagas disease.

The kDNA-mutated chicken model suggests a parasite-induced familial genetic disease; the genotype modifications in association with the autoimmune rejection of the heart originate from disruption of the tolerance mechanism of ‘self’ recognition by the host immune system [97]. The genetic control of immune tolerance present in healthy chickens is impaired in kDNA-mutated birds with rampant inflammatory cardiomyopathy. Autoimmunity plays a pivotal role in a substantial proportion of patients with genetically driven inflammatory cardiomyopathy of unknown etiology [100]–[102]. Also, the high risk reported for familial occurrence of cardiomyopathy in first-generation relatives suggests disruption of immune response mechanisms early in the development of the disease, and the identification of inflammatory infiltrates in the heart is an ominous sign of poor disease outcome. Further studies of chromosome skewing and instability-generated long range signaling interactions [60, 61, 74, and 75] are required to understand the genetically-induced mechanism of rupture of immune tolerance, and to explaining the attenuation of heart disease in descendents with genetic modifications.

Genetic mutations may generate myocarditis and dilated cardiomyopathy in humans, and the identification of underlying mutations, susceptibility and modifier genes are indispensable for development of new therapies [98, 101, and 102]. Experimental treatment of the inflammatory autoimmune cardiomyopathy in kDNA-mutated chickens may require drug suppression of bone marrow progenitor of specific T-cell phenotype infiltrating the myocardium, and transplantation of histocompatible healthy bone marrow to prevent the rejection of self-tissue. Thus, investigation in the congenic chicken model is underway, aimed at the inhibition of inflammatory cardiomyopathy by passive transfer of healthy, naïve bone marrow cells, and, consequently, an effective therapy for Chagas disease.

## Supporting Information

Figure S1 ***Trypanosoma cruzi* infection established in *Gallus gallus* embryo.** The dividing *T. cruzi* amastigotes are detected in the cytoplasm of 5 day-old chicken embryo mesodermal and endodermal cells by the specific fluorescein-labeled anti-*T. cruzi* antibody and by the X-gal stained β-galactosidase-expressing parasites. A) The *T. cruzi* trypomastigote silhouette is depicted by the fluorescein labeled specific antibody (dilution 1∶128 in PBS, pH 7.4) from a Chagas patient. Insert shows a fluorescein labeled amastigote parasitic form. B) Hematoxilin and eosin stained mesodermal and endodermal tissue from a control chicken embryo (magnification 100X). C) The control chicken embryo tissue section does not stain by the treatment with the fluorescein labeled specific anti-*T. cruzi* antibody (dilution 1∶32). D) *T. cruzi* growth in endodermal and mesodermal cells from a chicken embryo is shown by the specific fluorescein labeled antibody from a Chagas patient. E) Paraffin-embedded section showing the *T. cruzi* infected cells colocalized in the same embryo mesodermal and endodermal tissues by the X-gal stained β-galactosidase-expressing parasites.
(0.57 MB TIF)

Figure S2 **Pedigree showing lineage of chickens with *Trypanosoma cruzi* kDNA minicircle sequence integrated into the genome.** The parental hatched from *T. cruzi* inoculated egg vertically transferred the kDNA mutations to progeny F1 to F3. Asterisks refer to chickens subjected to tpTAIL-PCR, whose amplicons were cloned and sequenced.
(0.07 MB TIF)

Figure S3 **Direct detection of *Trypanosoma cruzi* kDNA in *Gallus gallus* parental and progeny.** Southern hybridizations of (A) *Eco*RI and (B) *Mbo*I digests of chicken heart DNA separated through a 0.8% agarose gel, blotted and hybridized with whole minicircle probe. *T. cruzi* mitochondrial kDNA (Tc) and uninfected chicken heart DNA were used as positive and negative controls (c).
(1.64 MB TIF)

Figure S4 **The *tp*TAIL-PCR control and validation experiments.** A) Template DNA from a kDNA-mutated bird subjected to *tp*TAIL-PC with different combination of kDNA primers with *Gg*1-to-*Gg*6 primers sets in subsequent amplifications throughout three cycles, showing an increasing specificity (few bands) after hybridization with radio labeled kDNA probe on blots of 1% agarose gel. B) The *tp*TAIL-PCR unique specificity shown by a mix of *T. cruzi* kDNA with control chicken DNA. The amplification products hybridized with the radio labeled kDNA probe, which were cloned and sequenced, and revealed kDNA minicircle only. C) The control *tp*TAIL-PCR amplification products from control chicken did not hybridize with the specific kDNA probe.
(2.46 MB TIF)

Figure S5 **Vertical transfer of kDNA minicircle from *Trypanosoma cruzi* from parental to *Gallus gallus* progeny.** A) Alignments of chimeras host DNA-kDNA minicircle transferred from rooster F0 (AY237306) to hen F1 (FN600557), locus NW_001471687.1 at chromosome 4. B) Ibid, from hen F1 (FN598991) to sibling F2 (FR681733), locus NW_001471679.1 at chromosome 1.
(3.41 MB TIF)

Figure S6 **Microhomologies present in *Trypanosoma cruzi* kDNA minicircles and in the *Gallus gallus* genome.** A) Major CA-rich consensus sequence. B) Minor consensus.
(1.69 MB TIF)

Figure S7 **Chagas-like dilated inflammatory cardiomyopathy in a F2 chicken with kDNA mutation in the dystrophin gene.** A) Dilated heart occupying most of the thoracic cavity (heart weight  = 16 g). B) Dark round mononuclear cells infiltrates and destroys the myocardium of the kDNA-mutated hen 20 (Table S2). C) Normal heart size (weight 7 g) of a 10-month-old control chicken. D) Normal histology of a control chicken heart.
(0.74 MB TIF)

Table S1 Lateral transfer of *Trypanosoma cruzi* kDNA minicircle into *Gallus gallus* genome and its vertical inheritance by progeny.
(0.09 MB DOC)

Table S2 Integration of *Trypanosoma cruzi* kDNA minicircle sequences into coding regions of *Gallus gallus*.
(0.06 MB DOC)

Table S3 Chimera protein sequences translated from ORFs formed by *Trypanosoma cruzi* mitochondrial kDNA minicircles inserted in the *Gallus gallus* genome^*^.
(0.02 MB DOCX)
