# Supplementary figures and images for: Trypanosoma cruzi in the Chicken Model: Chagas-Like Heart Disease in the Absence of Parasitism
Source: PLoS Negl Trop Dis. 2011 Mar 29;5(3):e1000. doi: 10.1371/journal.pntd.0001000 (PMC3066158; doi:10.1371/journal.pntd.0001000)

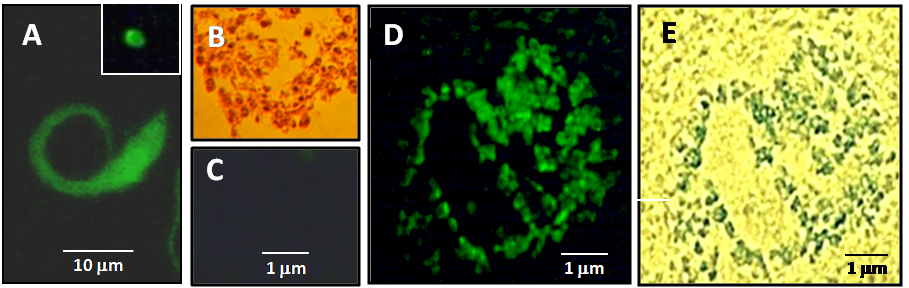

Supplement: Figure S1 — Trypanosoma cruzi infection established in Gallus gallus embryo. The dividing T. cruzi amastigotes are detected in the cytoplasm of 5 day-old chicken embryo mesodermal and endodermal cells by the specific fluorescein-labeled anti-T. cruzi antibody and by the X-gal stained β-galactosidase-expressing parasites. A) The T. cruzi trypomastigote silhouette is depicted by the fluorescein labeled specific antibody (dilution 1∶128 in PBS, pH 7.4) from a Chagas patient. Insert shows a fluorescein labeled amastigote parasitic form. B) Hematoxilin and eosin stained mesodermal and endodermal tissue from a control chicken embryo (magnification 100X). C) The control chicken embryo tissue section does not stain by the treatment with the fluorescein labeled specific anti-T. cruzi antibody (dilution 1∶32). D) T. cruzi growth in endodermal and mesodermal cells from a chicken embryo is shown by the specific fluorescein labeled antibody from a Chagas patient. E) Paraffin-embedded section showing the T. cruzi infected cells colocalized in the same embryo mesodermal and endodermal tissues by the X-gal stained β-galactosidase-expressing parasites. (0.57 MB TIF) [file pntd.0001000.s001.tif]

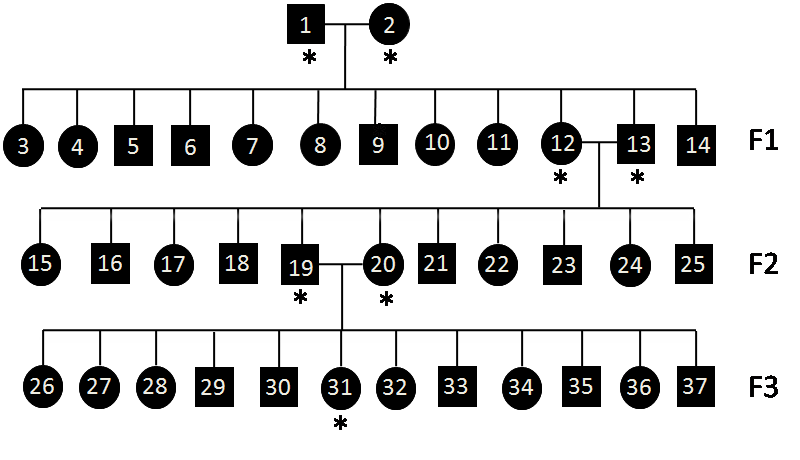

Supplement: Figure S2 — Pedigree showing lineage of chickens with Trypanosoma cruzi kDNA minicircle sequence integrated into the genome. The parental hatched from T. cruzi inoculated egg vertically transferred the kDNA mutations to progeny F1 to F3. Asterisks refer to chickens subjected to tpTAIL-PCR, whose amplicons were cloned and sequenced. (0.07 MB TIF) [file pntd.0001000.s002.tif]

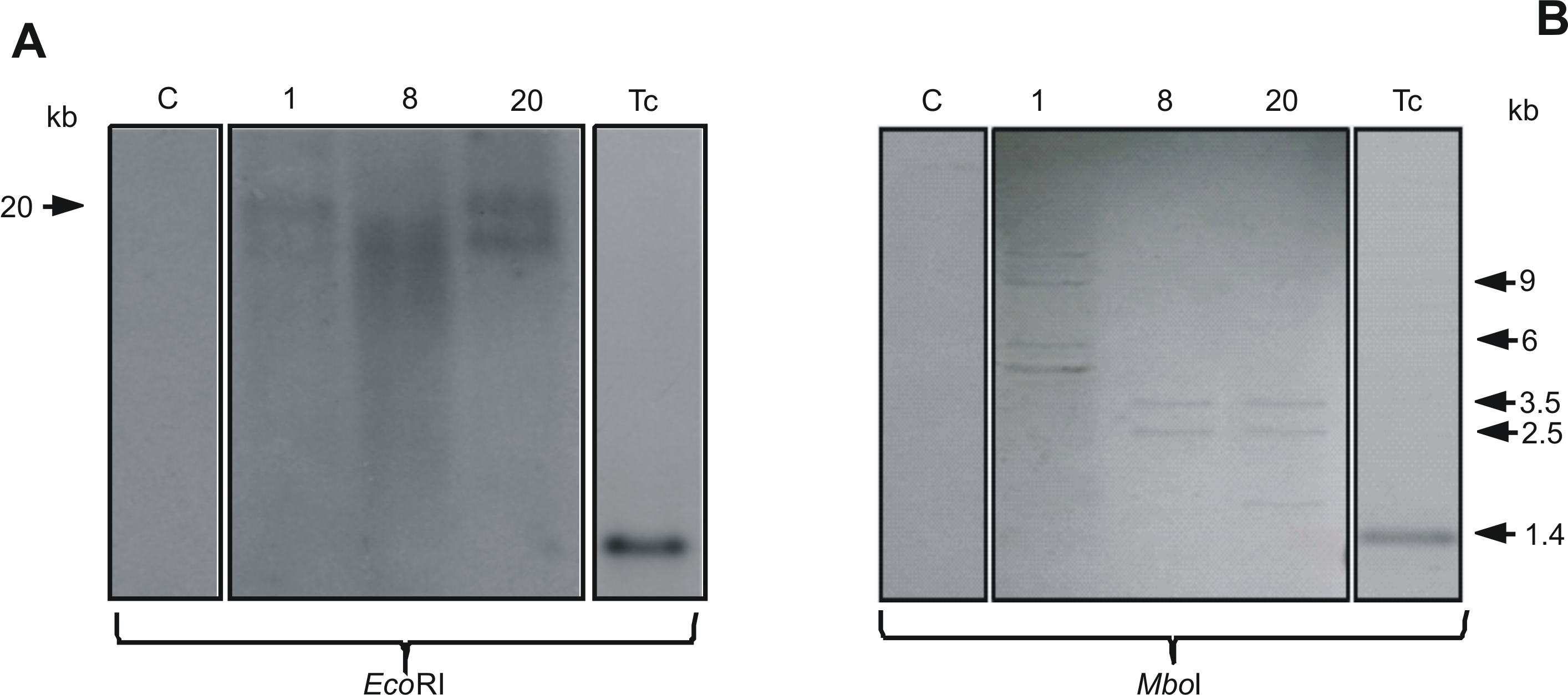

Supplement: Figure S3 — Direct detection of Trypanosoma cruzi kDNA in Gallus gallus parental and progeny. Southern hybridizations of (A) EcoRI and (B) MboI digests of chicken heart DNA separated through a 0.8% agarose gel, blotted and hybridized with whole minicircle probe. T. cruzi mitochondrial kDNA (Tc) and uninfected chicken heart DNA were used as positive and negative controls (c). (1.64 MB TIF) [file pntd.0001000.s003.tif]

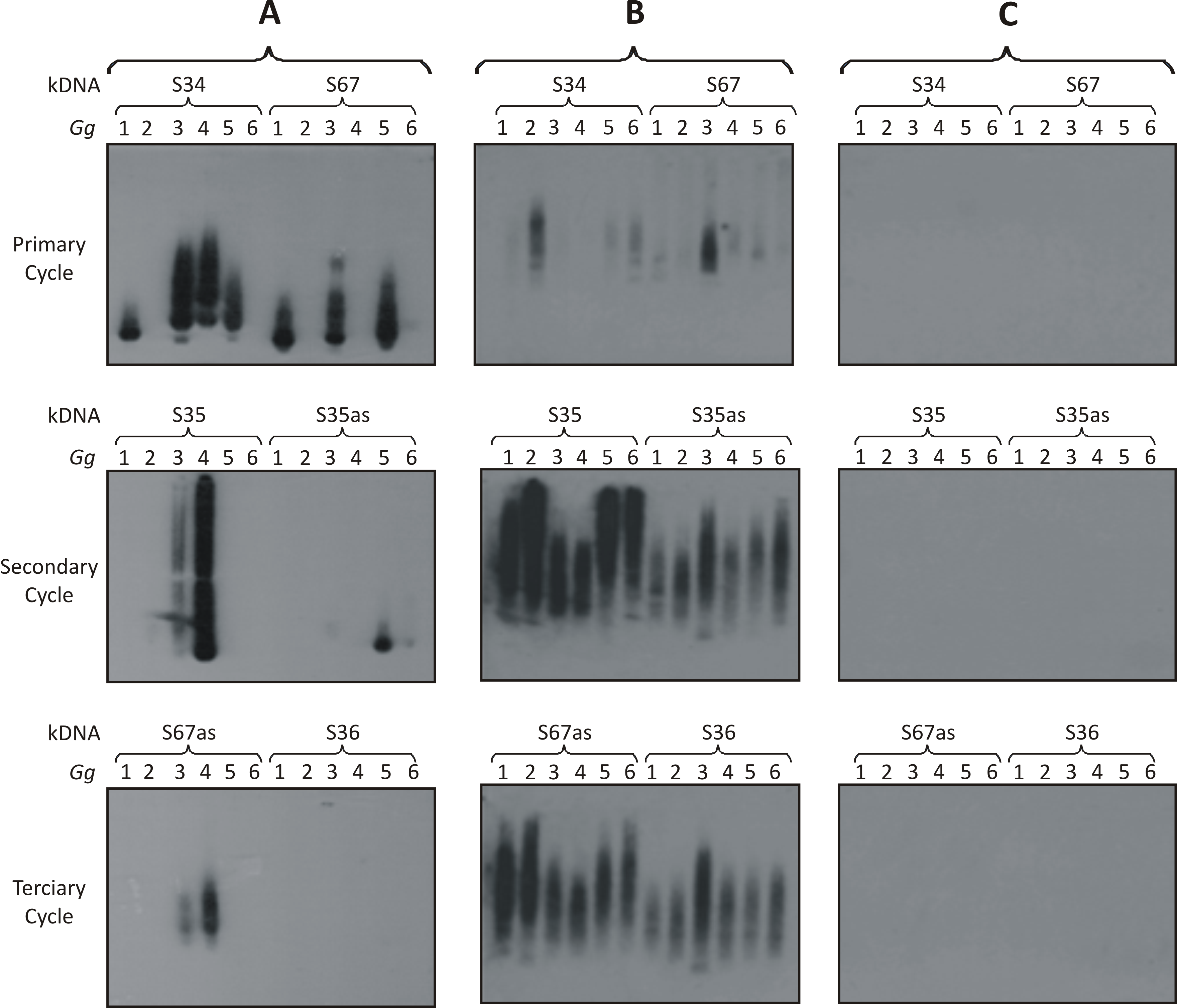

Supplement: Figure S4 — The tpTAIL-PCR control and validation experiments. A) Template DNA from a kDNA-mutated bird subjected to tpTAIL-PC with different combination of kDNA primers with Gg1-to-Gg6 primers sets in subsequent amplifications throughout three cycles, showing an increasing specificity (few bands) after hybridization with radio labeled kDNA probe on blots of 1% agarose gel. B) The tpTAIL-PCR unique specificity shown by a mix of T. cruzi kDNA with control chicken DNA. The amplification products hybridized with the radio labeled kDNA probe, which were cloned and sequenced, and revealed kDNA minicircle only. C) The control tpTAIL-PCR amplification products from control chicken did not hybridize with the specific kDNA probe. (2.46 MB TIF) [file pntd.0001000.s004.tif]

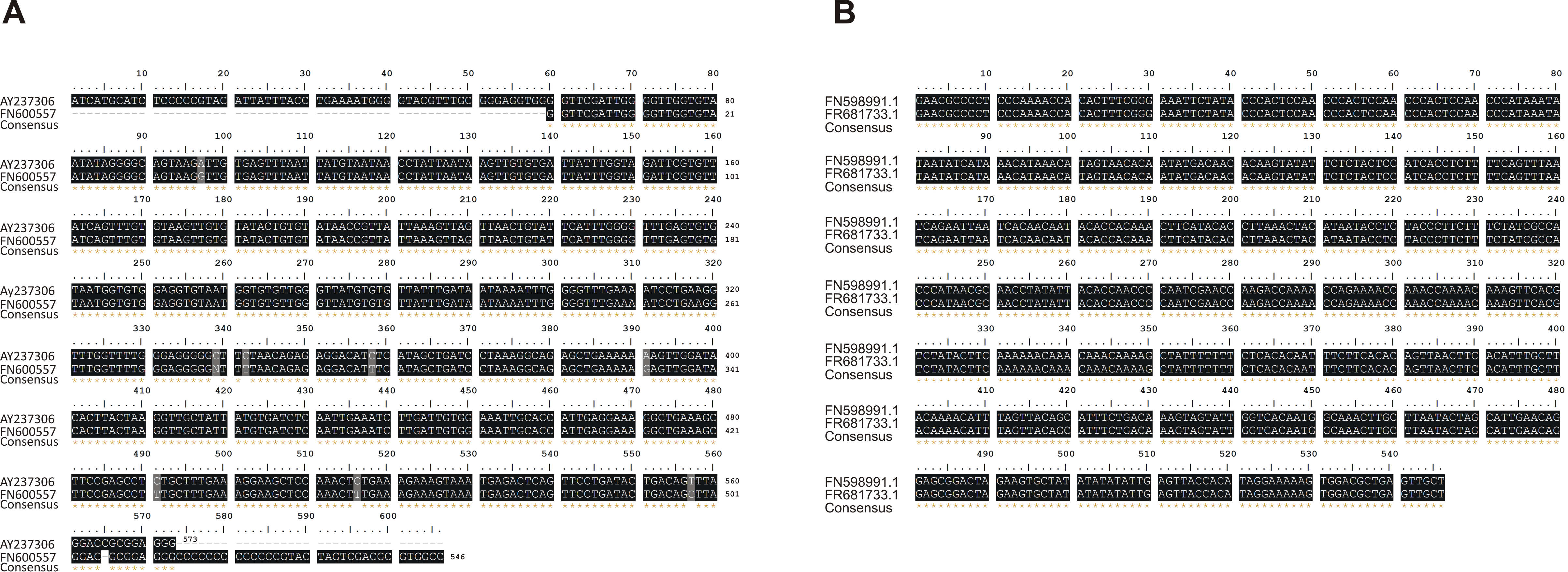

Supplement: Figure S5 — Vertical transfer of kDNA minicircle from Trypanosoma cruzi from parental to Gallus gallus progeny. A) Alignments of chimeras host DNA-kDNA minicircle transferred from rooster F0 (AY237306) to hen F1 (FN600557), locus NW_001471687.1 at chromosome 4. B) Ibid, from hen F1 (FN598991) to sibling F2 (FR681733), locus NW_001471679.1 at chromosome 1. (3.41 MB TIF) [file pntd.0001000.s005.tif]

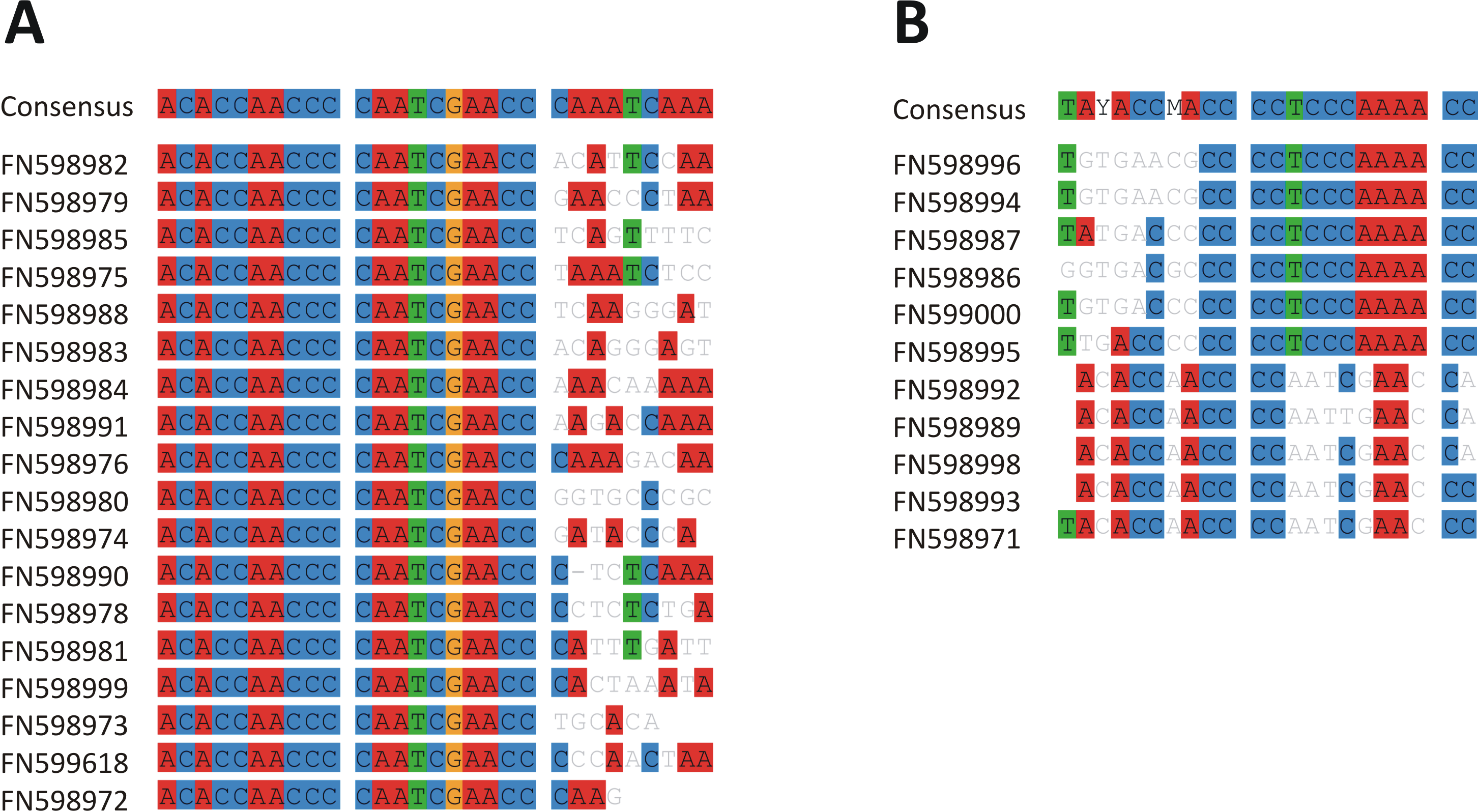

Supplement: Figure S6 — Microhomologies present in Trypanosoma cruzi kDNA minicircles and in the Gallus gallus genome. A) Major CA-rich consensus sequence. B) Minor consensus. (1.69 MB TIF) [file pntd.0001000.s006.tif]

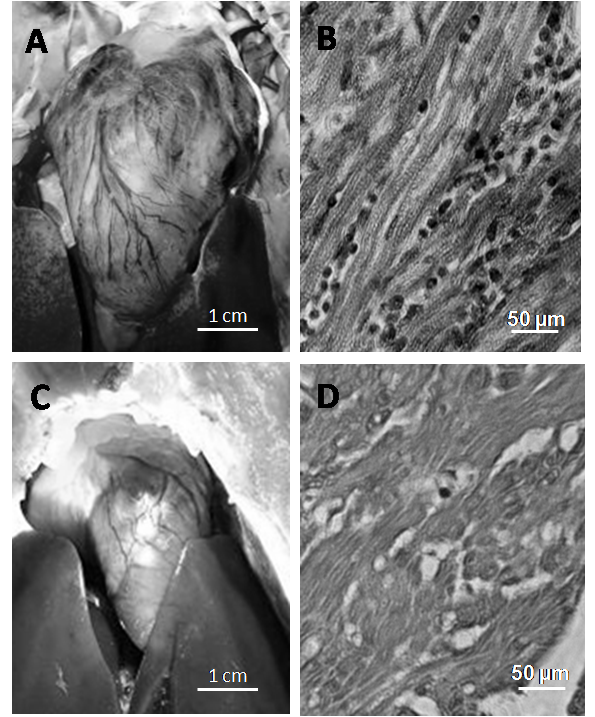

Supplement: Figure S7 — Chagas-like dilated inflammatory cardiomyopathy in a F2 chicken with kDNA mutation in the dystrophin gene. A) Dilated heart occupying most of the thoracic cavity (heart weight = 16 g). B) Dark round mononuclear cells infiltrates and destroys the myocardium of the kDNA-mutated hen 20 (Table S2). C) Normal heart size (weight 7 g) of a 10-month-old control chicken. D) Normal histology of a control chicken heart. (0.74 MB TIF) [file pntd.0001000.s007.tif]
