# Supplementary material for: Trypanosoma cruzi in the Chicken Model: Chagas-Like Heart Disease in the Absence of Parasitism
Source: PLoS Negl Trop Dis. 2011 Mar 29;5(3):e1000. doi: 10.1371/journal.pntd.0001000 (PMC3066158; doi:10.1371/journal.pntd.0001000)
Supplement: Table S1 — Lateral transfer of Trypanosoma cruzi kDNA minicircle into Gallus gallus genome and its vertical inheritance by progeny. (0.09 MB DOC) [file pntd.0001000.s008.doc]

| **Table S1**. Lateral transfer of *Trypanosoma cruzi* kDNA minicircle into *Gallus gallus* genome and its vertical inheritance by progeny | | | | | | | | | | |
| --- | --- | --- | --- | --- | --- | --- | --- | --- | --- | --- |
|  | |  | |  | | | | | | |
| Chicken | Accession # | | kDNA/Host DNA | | kDNA *E-value* | Chicken DNA *E-value* | Intermediate sequence | Chromosome | Locus | Description |
| 1 | AY237306 | | 1-278/ 273-504 | | 3e-04 | 4e-100 | GAGGGG | 4 | NW_001471687.1 | ND |
| 1 | FN598971 | | 85-420/1-100 | | 1.6e-139 | 2e-38 | ACACCAACCCCAATCGAACCCC | 21 | NW_001471574.1 | Similar to eukaryotic translation initiation factor 4 gamma |
| 1 | FN598972 | | 1-294/ 274-430 | | 6.1e-145 | 1e-55 | ACACCAACCCCAATCGAACCCCAACCAC | 14 | NW_001471454.1 | [Similar to PI-3-kinase-related kinase SMG-1](http://www.ncbi.nlm.nih.gov/entrez/viewer.fcgi?val=118098117&db=Nucleotide&from=8531284&to=8591866&view=gbwithparts&RID=FSFTUCND01N) |
| 1 | FN598973 | | 1-277/ 254-574 | | 5e-129 | 9e-153 | ACACCAACCCCAATCGAACCTGCACA | 25 | NW_001471593.1 | ND |
| 1 | FN598974 | | 1-297/ 277-617 | | 2e-141 | 3e-160 | ACACCAACCCCAATCGAACCG | 1 | NW_001471549.1 | ND |
| 1 | FN598975 | | 1-288/ 268-528 | | 1e-06 | 9e-115 | ACACCAACCCCAATCGAACCTAA | 2 | NW_001471655.1 | CR1 similar to KIAA1882 protein |
| 1 | FN598976 | | 1-246/ 234-285 | | 9e-09 | 5e-13 | AACCCCAAAGACAA | 11 | NW_001471435.1 | Hypothetical protein - PEPD peptidase D |
| 2 | FN598977 | | 1-324/ 306-595 | | 5e-135 | 3e-133 | GCATCTCC | 1 | NW_001471556.1 | ND |
| 12 | FN598978 | | 1-298/ 277-418 | | 1e-146 | 1e-53 | ACACCAACCCCAATCGAACCCC | 4 | NW_001471681.1 | Hypothetical protein - GPC3 glypican 3 |
| 12 | FN598979 | | 1-353/ 327-480 | | 1e-85 | 2e-67 | ACACCAACCCCAATCGAACC | 22 | NW_001471575.1 | [Similar to tumor endothelial marker 8](http://www.ncbi.nlm.nih.gov/entrez/viewer.fcgi?val=118101334&db=Nucleotide&from=153667&to=245489&view=gbwithparts&RID=7YF4TKAR014) |
| 12 | FN598980 | | 1-304/ 284-379 | | 3e-148 | 3e-29 | ACACCAACCCCAATCGAACC | 11 | NW_001471432.1 | [Vac14 homolog](http://www.ncbi.nlm.nih.gov/entrez/viewer.fcgi?val=118096260&db=Nucleotide&from=876117&to=928929&view=gbwithparts&RID=7YEZNPYU015) |
| 12 | FN598981 | | 1-288/ 266-497 | | 3e-143 | 6e-105 | ACACCAACCCCAATCGAACCCA | 1 | NW_001471554.1 | [Similar to RhoGTPase regulating protein variant ARHGAP20-1ad](http://www.ncbi.nlm.nih.gov/entrez/viewer.fcgi?val=118085257&db=Nucleotide&from=17794265&to=17853185&view=gbwithparts&RID=7YEUMK42014) |
| 12 | FN598982 | | 1-289/ 267-644 | | 4e-143 | 0 | ACACCAACCCCAATCGAACCACA | 14 | NW_001471454.1 | [Similar to PI-3-kinase-related kinase SMG-1](http://www.ncbi.nlm.nih.gov/entrez/viewer.fcgi?val=118098117&db=Nucleotide&from=8531284&to=8591866&view=gbwithparts&RID=7YENTU34012) |
| 12 | FN598983 | | 1-286/ 264-426 | | 2e-04 | 8e-51 | ACACCAACCCCAATCGAACCACA | 4 | NW_001471685.1 | ND |
| 12 | FN598984 | | 1-265/ 244-974 | | 9e-08 | 0 | ACACCAACCCCAATCGAACCA | 5 | NW_001471707.1 | ND |
| 12 | FN598985 | | 1-280/ 270-494 | | 2e-133 | 6e-99 | ACACCAACCCCAATCGAACC | 8 | NW_001471740.1 | ND |
| 12 | FN598986 | | 133-423/ 1-154 | | 1e-140 | 6e-59 | ACGCCCCCTCCCAAAACCAA | 3 | NW_001471679.1 | ND |
| 12 | FN598987 | | 138-431/ 1-162 | | 4e-147 | 2e-58 | AGACCCCCCTCCCCAAAACCACAC | 2 | NW_001471633.1 | Similar to src kinase-associated phosphoprotein 55-related protein |
| 12 | FN600557 | | 1-345/ 332-563 | | 3e-04 | 1e-101 | GAGGGGGCTTCTAA | 4 | NW_001471687.1 | ND |
| 12 | FN598991 | | 1-280/261-546 | | 6e-134 | 1e-58 | ACACCAACCC CAATCGAACCAA | 1 | NW_001471534.1 | [Dystrophin](http://www.ncbi.nlm.nih.gov/entrez/viewer.fcgi?val=118084132&db=Nucleotide&from=13558559&to=14559325&view=gbwithparts&RID=FSMVDSYW01S) |
| 13 | FN598988 | | 1-297/ 274-558 | | 1e-143 | 4e-102 | ACACCAACCCCAATCGAACCTCAA | 2 | NW_001471637.1 | [Similar to Adenylate cyclase type 1](http://www.ncbi.nlm.nih.gov/entrez/viewer.fcgi?val=118086413&db=Nucleotide&from=1410631&to=1586257&view=gbwithparts&RID=CDP3MR9B016) |
| 13 | FN598989 | | 136-435/ 1-151 | | 6e-07 | 5e-58 | CAACCCCAATCGAACC | 13 | NM_001001613.1 | CD74 molecule, major histocompatibility complex, class II invariant chain (CD74) |
| 13 | FN598990 | | 1-277/ 248-960 | | 1e-130 | 0 | ACACCAACCCCAATCGAACCCC | 2 | NW_001471639.1 | Zinc finger protein 516 |
| 13 | FN598992 | | 257-532/ 1-274 | | 1e-135 | 1e-120 | ACACCAACCCCAATCGAACC | 7 | NW_001471729.1 | Amyotrophic lateral sclerosis 2 (juvenile) chromosome region, candidate 2 |
| 13 | FN598993 | | 170-457/ 1-190 | | 3e-143 | 9e-78 | ACACCAACCCCAATCGAACC | 3 | NW_001471679.1 | ND |
| 19 | FN598994 | | 309-599/ 1-336 | | 3e-133 | 4e-85 | GAACGCCCCTCCCAAAACCAAAA | 1 | NW_001471554.1 | Similar to putative breast epithelial stromal interaction protein - EPSTI1 epithelial stromal interaction 1 |
| 19 | FN598995 | | 389-630/ 1-412 | | 3e-145 | 0 | GACCCCCCCTCCCAAAACCACAC | 14 | NW_001471454.1 | ND |
| 19 | FN598996 | | 142-798/ 1-159 | | 2e-46 | 4e-64 | GACCCCCCCTCCCAAAACCA | 9 | NW_001471743.1 | ND |
| 20 | FN598997 | | 1-338/ 325-610 | | 5e-28 | 2e-150 | AACGCCCCTCCC | 4 | NW_001471681.1 | [Dachshund homolog 2](http://www.ncbi.nlm.nih.gov/entrez/viewer.fcgi?val=118089733&db=Nucleotide&from=8338908&to=8605684&view=gbwithparts&RID=FUE3VNA001S) |
| 20 | FN598998 | | 406-696/ 1-423 | | 7e-143 | 0 | CCAACCCCAATCGAACC | 1 | NW_001471554.1 | [Similar to spinal cord-derived growth factor-B](http://www.ncbi.nlm.nih.gov/entrez/viewer.fcgi?val=118085257&db=Nucleotide&from=20229293&to=20366942&view=gbwithparts&RID=FSJMYEXT01S) |
| 20 | FN598999 | | 1-297/ 265-496 | | 2e-140 | 1e-99 | ACACCAACCCCAATCGAACCCAC | 1 | NW_001471529.1 | ND |
| 20 | FR681733 | | 1-280/261-546* | | 6e-134 | 1e-58 | ACACCAACCC CAATCGAACCAA | 1 | NW_001471534.1 | [Dystrophin](http://www.ncbi.nlm.nih.gov/entrez/viewer.fcgi?val=118084132&db=Nucleotide&from=13558559&to=14559325&view=gbwithparts&RID=FSMVDSYW01S) |
| 31 | FN599000 | | 426-711/ 1-448 | | 2e-140 | 0 | GACCCCCCCTCCCAAAACCAAA | 3 | NW_001471679.1 | [Similar to protein kinase C epsilon](http://www.ncbi.nlm.nih.gov/entrez/viewer.fcgi?val=118088167&db=Nucleotide&from=9586860&to=9862683&view=gbwithparts&RID=7Z9KUBSK01R) |
| 31 | FN599618 | | 260-358/ 1-297 | | 1,7e-3 | 1e ^-135^ | CAACCCCAAGCCAACCCAACCACAC | 3 | NW_001471673 | ND |

.
