# Supplementary material for: Trypanosoma cruzi in the Chicken Model: Chagas-Like Heart Disease in the Absence of Parasitism
Source: PLoS Negl Trop Dis. 2011 Mar 29;5(3):e1000. doi: 10.1371/journal.pntd.0001000 (PMC3066158; doi:10.1371/journal.pntd.0001000)
Supplement: Table S2 — Integration of Trypanosoma cruzi kDNA minicircle sequences into coding regions of Gallus gallus. (0.06 MB DOC) [file pntd.0001000.s009.doc]

**Table S2.**  Integration of *Trypanosoma cruzi* kDNA minicircle sequences into coding regions of *Gallus gallus*

| *Gallus gallus* | Parenthood/  Gender | Accession # | Locus | Chromosome | Gene Function* |
| --- | --- | --- | --- | --- | --- |
| 1 | F0/M | FN598971 | NW_001471574.1 | 21 | eIF4F protein complex involved in recognition of the mRNA cap, ATP-dependent unwinding of 5'-terminal secondary structure and recruitment of mRNA to the ribosome. |
| 1 | F0 M | FN598972 | NW_001471454.1 | 14 | Serine/threonine protein kinase (PI-3K) involved in mRNA surveillance and genotoxic stress response. Plays a role in nonsense-mediated decay of mRNAs by phosphorylating UPF1/RENT1. Acts as part of the SMG1C complex, a mRNA surveillance complex that recognizes and degrades mRNAs containing premature translation termination codons (PTCs). Acting as stress-activated protein kinase can phosphorylate TP53/p53 after cellular exposure to genotoxic stress. Its depletion leads to spontaneous DNA damage. |
| 1 | F0/M | FN598975 | NW_001471655.1 | 2 | NF-kappa-B activator through increased phosphorylation of the IKK complex. May function in immune system cells differentiation. |
| 1 | F0/M | FN598976 | NW_001471435.1 | 11 | Hypotetical gene. Molecular function inferred: aminopeptidase activity, manganese ion binding, metalloexopeptidase activity. |
| 12 | F1/M | FN598978 | NW_001471681.1 | 4 | GPC3 glypican involved in suppression/modulation of growth of mesodermal tissues and organs and tumor predisposition. |
| 12 | F1/M | FN598979 | NW_001471575.1 | 22 | Plays a role in cell attachment and migration. Interacts with extracellular matrix proteins and with the actin cytoskeleton. Mediates adhesion of cells to type 1 collagen and gelatin, promotes cell spreading and reorganization of the actin cytoskeleton. |
| 12 | F1/M | FN598980 | NW_001471432.1 | 11 | The PI(3,5)P2 regulatory complex regulates synthesis and turnover of phosphatidylinositol-3,5-bisphosphate (PtdIns(3,5)P2). Acts as a positive activator of PIK5 kinase activity. |
| 12 | F1/M | FN598981 | NW_001471554.1 | 1 | GTPase activator for the Rho-type GTPases by converting them to an inactive GDP-bound state. Translocation t(X;11)(q21;q23) with BRWD3 disrupts both genes. |
| 12 | F1/M | FN598982 | Nw_001471454.1 | 14 | PI-3 kinase related to kinase SMG-1 |
| 12 | F1/M | FN598987 | NW_001471633.1 | 2 | Involved in B-cell and macrophage adhesion processes. In B-cells, may act by coupling the B-cell receptor (BCR) to integrin activation. May play a role in src signaling pathway. |
| 13 | F1/F | FN598988 | NW_001471637.1 | 2 | This gene encodes a form of adenylate cyclase expressed in brain. A similar protein in mouse is involved in pattern formation of the brain. |
| 13 | F1/F | FN598989 | NM_001001613.1 | 13 | Major histocompatibility complex |
| 13 | F1/F | FN598990 | NW_001471639.1 | 2 | Involved in transcriptional regulation. |
| 13 | F1/F | FN598991 | NW_001471534.1 | 1 | Play a role in anchoring the cytoskeleton to the plasma membrane. |
| 13 | F1/F | FN598992 | NW_001471729.1 | 7 | Regulates transcription activation factors and regulation of p53 pathway. |
| 19 | F2/M | FN598994 | NW_001471554.1 | 1 | Indeterminate function. Expressed in blood mononuclear cells from patients with systemic lupus erythematosus (SLE). |
| 20 | F2/F | FN598997 | NW_001471681.1 | 4 | Transcription factor involved in regulation of organogenesis. |
| 20 | F2/F | FN598998 | NW_001471554.1 | 1 | Potent mitogen for cells of mesenchymal origin. Binding of this growth factor to its affinity receptor elicits a variety of cellular responses. Induces macrophage recruitment, and blood vessel maturation during angiogenesis. |
| 31 | F3/F | FN599000 | NW_001471679.1 | 3 | Calcium-independent, phospholipid-dependent, serine-threonine-specific (PKC) activated by diacylglycerol which in turn phosphorylates a range of cellular proteins. PKC also serves as receptor for phorbolesters. |

* Gene definition made by UniProt ([www.uniprot.org](http://www.uniprot.org/))
