# Supplementary material for: Trypanosoma cruzi in the Chicken Model: Chagas-Like Heart Disease in the Absence of Parasitism
Source: PLoS Negl Trop Dis. 2011 Mar 29;5(3):e1000. doi: 10.1371/journal.pntd.0001000 (PMC3066158; doi:10.1371/journal.pntd.0001000)
Supplement: Table S3 — Chimera protein sequences translated from ORFs formed by Trypanosoma cruzi mitochondrial kDNA minicircles inserted in the Gallus gallus genome*. (0.02 MB DOCX) [file pntd.0001000.s010.docx]

**Table S3.** Chimera Protein Sequences Translated from ORFs Formed by *Trypanosoma cruzi* Mitochondrial kDNA Minicircles Inserted in the *Gallus gallus* Genome*

| **Chicken** | **EMBL** | **ORFs translated putative proteins** | **Locus** | **BlastX** | ***E-value*** |
| --- | --- | --- | --- | --- | --- |
| **1** | AY237306 | Not found |  |  |  |
| 1 | FN598971 | MCGICTCERNDVGKIVRVVVCYILTIVI  MDTDKMKYRMCIDSLIGMYCMDLNM | -  CAR63082.1 | No significant similarity  Hypothetical protein | ^-^  4e-08 |
| 1 | FN598972 | Not found |  |  |  |
| 1 | FN598973 | MPLPKPQFSQNYRLPYRNQTYTILQTQPHTLPMQYKLYP | - | No significant similarity | - |
| 1 | FN598974 | MDIPSCGRVLKGVGIHITLFHCPGSWLGIGSIGVGVI  MWESFEGGWNSYHSLSLPRVLAGYRFDWGWCNISKSGYCILEL | - | No significant similarity  No significant similarity | - |
| 1 | FN598975 | MMITGLERLSCEDRLRELGLFSLEKRRLRGDLIAAFQYLKGAY  KQEGSKLFESVDNRRTRGNGFKLKEGRFRFDWGWCNI | AAA49027.1 | Reverse transcriptase *Gallus gallus* | 4e-29 |
| 1 | FN598976 | MHEFPPQKLTPTPTLSGLQLPNKSITAYS  MHLPRTLWFWEGRSNLGRKIHASHPYIMVLGGA  MRLHKKCLRELVFGFDWGWCNIDGCVEVVNIMSRL | CAR63087.1  CAR63104.2  - | Hypothetical protein  Ibid  No significant similarity | 2e-06  1e-11  - |
| 2 | FN598977 | MYGEMPQVVGFSPAAGEKGGEGINAGGEMDGEAVSVGYSTIRKIGI  METCTCSGGPRWPICRFLYIPIFLIVLYPTDTASPSISPPALIPS  PPFSPAAGLNPTTISPYIICLF | -  - | No significant similarity  No significant similarity | -  - |
| 12 | FN598978 | MWCFLNLWFQFQAVEMIVCPACVTAVREGFDWGWCNIERWGIEIWMYISFVILMMCIV  MGNVVLFESLVSVSGCRDDSLSCVCYCCQRGVRLGLV | -  - | No significant similarity  No significant similarity | -  - |
| 12 | FN598979 | MKNGASVRVMVRVRFDWGWCIQRFNSFLVYSWEGACVSEPAIGLFKDSTSSWFSLGRGVQ IWARKIMHLTRTLYKGISPVHYSFGRGGQIWAGKFMHLPRTLWFWEGRSNLGRRIHASHP YIMVLGGG | CAR634104.2 | Hypothetical protein | 2e-22 |
| 12 | FN598980 | Not found |  |  |  |
| 12 | FN598981 | MYPKSTHPILHQPQSNPFDCHGSLLPSGTYTPYSSDLI | CAR63070.1 | Hypothetical protein | 6e-04 |
| 12 | FN598982 | MVQPTAQFCQPVSLKGKTSLNPLINWEPLGQREQGAVELPGRLECGSIGVGVI | - | No significant similarity | - |
| 12 | FN598983 | Not found |  |  |  |
| 12 | FN598984 | MSVMYIHYGIKIMGCYCGSYTLKWVREI | - | No significant similarity | - |
| 12 | FN598985 | MLFSLSLLATLAMPWESGLWQNQTLEKHDRMKTEVRLGLV | - | No significant similarity | - |
| 12 | FN598987 | MNAPFQKTCACFRVFNISLPRSVWCLAVPVDHSCMTPPPKTTLSGNSQPYSHPKYTK | - | No significant similarity | - |
| 12 | FN598989 | MYMIEVCMYVWMDGMVWCIMVIVRIEKCSGSEMNSFLIIMYLYRYLINVVCCWIRCGWCI | - | No significant similarity | - |
| 12 | FN600557 | Not found |  |  |  |
| 12 | FN598991 | MGWSGLEWVGVGIEFPESVVLGGA | - | No significant similarity | - |
| 13 | FN598988 | Not found |  |  |  |
| 13 | FN598989 | Not found |  |  |  |
| 13 | FN598991 | MGWSGLEWVGVGIEFPESVLGGA | - | No significant similarity | - |
| 13 | FN598992 | MKVYLKFVNRIIKVCYNCLNKGVVVACGFVVDSGYIMRVEISKMLVLGGAF | - | No significant similarity | - |
| 13 | FN598993 | MCRFRIHLELSLKFICIIEHVKITVQHGVVDSSVDLYVSIC  MYPKSTHPILHQPQSNPNKQLIMELLKIAFAHLC | -  - | No significant similarity  No significant similarity | -  - |
| 19 | FN598994 | Not found |  |  |  |
| 19 | FN598995 | MIAFDPPLPKPHFREILNLTHTLSIPNNSNHNHHNYHTIPHTILSKIILYK | - | No significant similarity | - |
| 19 | FN598996 | MYLLYNLSHSVLNNITMTTQNIIHHKISILYSATYYRPTLYYTNPNRT  MQTRENIHGLCERPSQNQTLDFQPTTN  D MHDSGPNLNAPPKTEFSGISYTHFTIVTTTTSHLTPTNQKLNYKV | -  -  CAR63077.1 | No significant similarity  No significant similarity  Hypothetical protein | -  -  - |
| 20 | FN598997 | MYIKMDKRRDETLRENVEILFIVFS | - | No significant similarity | - |
| 20 | FN598998 | MDTDKMKYRMCIDSLIGMYCMDLNM  MYISHTSHIQIHTIHTNQTINTHSIFHLISIHSNKPYITPTPIEPSYPNSNSPPAQSWP | CAR63082.1  CAR63082.1 | Hypothetical protein  Hypothetical protein | -  - |
| 20 | FN598900 | MRCRAFTNNTQGLSAPPVQLVNENKNENTLQSRTASPMTTFYSVSCAVTRDKMIVTPPPKTKFS | - | No significant similarity | - |
| 20 | FR681733 | MGWSGLEWVGVGIEFPESVLGGA | - | No significant similarity | - |
| 31 | FN599000 | Not found |  |  |  |
| 31 | FN599618 | MVQFPQSRFQLRSHNSNLSKCIQLFFSSAFRISYEMSSLLKXPSQNQTFRIFKPQILLSN THNPTHHYTSTPLHTQTPNEYS  MGVHLSLLLLEGLVGGSIGVGVIMQLSGLEFSNQCELILKAE | -  - | No significant similarity  No significant similarity | -  - |

* ORFs derive* ORFs derived Chimera proteins formed in the kDNA integration events: Blue, kDNA; Green, *Gallus gallus* DNA.
